# Coevolution of Siglec-11 and Siglec-16 via gene conversion in primates

**DOI:** 10.1186/s12862-017-1075-z

**Published:** 2017-11-23

**Authors:** Toshiyuki Hayakawa, Zahra Khedri, Flavio Schwarz, Corinna Landig, Suh-Yuen Liang, Hai Yu, Xi Chen, Naoko T. Fujito, Yoko Satta, Ajit Varki, Takashi Angata

**Affiliations:** 1 Faculty of Arts and Science, Kyushu University, 744 Motooka, Nishi-ku, Fukuoka, 819-0395 Japan; 2 Departments of Medicine and Cellular & Molecular Medicine, Glycobiology Research and Training Center, University of California at San Diego, La Jolla, CA USA; 3 Institute of Biological Chemistry, Academia Sinica, 128 Section 2, Academia Road, Nangang District, Taipei, 11529 Taiwan; 4 Department of Chemistry, University of California at Davis, Davis, CA USA; 5 Department of Evolutionary Studies of Biosystems, SOKENDAI (Graduate University for Advanced Studies), Hayama, Kanagawa, Japan

**Keywords:** Coevolution, Sialic acid, Paired receptors, Gene conversion, Primates

## Abstract

**Background:**

Siglecs-11 and -16 are members of the sialic acid recognizing Ig-like lectin family, and expressed in same cells. Siglec-11 functions as an inhibitory receptor, whereas Siglec-16 exhibits activating properties. In humans, *SIGLEC11* and *SIGLEC16* gene sequences are extremely similar in the region encoding the extracellular domain due to gene conversions. Human *SIGLEC11* was converted by the nonfunctional *SIGLEC16P* allele, and the converted *SIGLEC11* allele became fixed in humans, possibly because it provides novel neuroprotective functions in brain microglia. However, the detailed evolutionary history of *SIGLEC11* and *SIGLEC16* in other primates remains unclear.

**Results:**

We analyzed *SIGLEC11* and *SIGLEC16* gene sequences of multiple primate species, and examined glycan binding profiles of these Siglecs. The phylogenetic tree demonstrated that gene conversions between *SIGLEC11* and *SIGLEC16* occurred in the region including the exon encoding the sialic acid binding domain in every primate examined. Functional assays showed that glycan binding preference is similar between Siglec-11 and Siglec-16 in all analyzed hominid species. Taken together with the fact that Siglec-11 and Siglec-16 are expressed in the same cells, Siglec-11 and Siglec-16 are regarded as paired receptors that have maintained similar ligand binding preferences via gene conversions. Relaxed functional constraints were detected on the *SIGLEC11* and *SIGLEC16* exons that underwent gene conversions, possibly contributing to the evolutionary acceptance of repeated gene conversions. The frequency of nonfunctional *SIGLEC16P* alleles is much higher than that of *SIGLEC16* alleles in every human population.

**Conclusions:**

Our findings indicate that Siglec-11 and Siglec-16 have been maintained as paired receptors by repeated gene conversions under relaxed functional constraints in the primate lineage. The high prevalence of the nonfunctional *SIGLEC16P* allele and the fixation of the converted *SIGLEC11* imply that the loss of Siglec-16 and the gain of Siglec-11 in microglia might have been favored during the evolution of human lineage.

**Electronic supplementary material:**

The online version of this article (10.1186/s12862-017-1075-z) contains supplementary material, which is available to authorized users.

## Background

Sialic acids are a family of nine-carbon sugars that are found at the outer end of glycan chains on the cell surface and secreted molecules in the deuterostome lineage, and play important roles as recognition components in cell–cell communication and host–pathogen interactions [1–3]. Siglecs (sialic acid-binding immunoglobulin superfamily lectins) are type I transmembrane proteins that recognize sialic acids and are mostly expressed in the cells involved in immunity [4–7]. Their extracellular regions consist of one V-set immunoglobulin-like domain (V-set domain) that is essential for sialic acid recognition, and variable numbers of C2-set immunoglobulin-like domains. Many Siglecs have signaling motifs in their cytoplasmic tail or transmembrane domain and induce signal transduction in cells.

Siglec-11 has inhibitory signaling motifs (immunoreceptor tyrosine-based inhibitory motifs: ITIMs) in its cytoplasmic tail and functions as a receptor that inhibits cell functions by recognizing sialic acids [8]. On the other hand, Siglec-16 has no signaling motifs, but activates cell functions by association with an adaptor molecule that contains immunoreceptor tyrosine-based activating motif (ITAM) [9]. Both Siglec-11 and Siglec-16 are expressed on macrophages, and probably regulate cell signaling [8, 9]. *SIGLEC11* and *SIGLEC16* genes are found in head-to-head orientation about 9 kb apart in the human genome [8, 9]. The sequences of the ~3-kb genomic regions containing exons encoding N-terminal region (V-set domain and C2-set domains) in these two genes are highly similar. This evolutionary kinship suggests that these Siglec genes originally emerged via a gene duplication event [8, 9]. Interestingly, the human *SIGLEC16* locus has functional and nonfunctional alleles within populations [9, 10]. The nonfunctional allele (*SIGLEC16P* allele) has a 4 base pair deletion in exon 2, and produces a very short protein (Siglec-16P), if any, lacking a transmembrane domain. We previously reported that *SIGLEC16P* converted the *SIGLEC11* gene about 1 million years ago (MYA) in the human lineage (*SIGLEC16P*→*SIGLEC11* gene conversion)[10], and the human *SIGLEC11* gene came to be expressed in brain microglia [11]. In addition, immunostaining using anti-Siglec-16 antibody suggested that human Siglec-16 is also expressed in brain microglia [9]. The human-specific *SIGLEC16P*→*SIGLEC11* gene conversion included ~240 bp of 5′ untranslated region of the gene [11], and a GATA-1-binding sequence, which is known as a repressor of BACE1 transcription in rat microglial cells [12], is modified by the *SIGLEC16P*→*SIGLEC11* gene conversion ([10]; see Additional file 1: Figure S1). Thus, it is possible that the brain expression of human Siglec-11 emerged via the *SIGLEC16P*→*SIGLEC11* gene conversion. The *SIGLEC16P*→*SIGLEC11* gene conversion is an example of a pseudogene (a nonfunctional allele) contributing to the evolution of a functional gene. Surprisingly, *SIGLEC16P* has persisted for over 3 million years (MYR) in the human lineage. Three hypotheses have been proposed to explain this [7, 10]. One is a gradual phasing out of Siglec-16 [7]. Siglec-11 shows neuroprotective effects such as inhibition of the production of proinflammatory mediators, and plays an important role in the immune function of microglia [13]. Siglec-16 activates immune and inflammatory responses in the brain microglia and may counteract the neuroprotective effect of Siglec-11. This unfavorable role of Siglec-16 in the brain might have resulted in the elimination of Siglec-16 in the human lineage. A second hypothesis proposes a balance between pathogenic pressure to retain Siglec-16 within populations and the elimination of Siglec-16 driven by its detrimental effects on immune activation [7]. The last hypothesis is hitchhiking on the adjacent *SIGLEC11* gene [10]. After the *SIGLEC16P*→*SIGLEC11* gene conversion, the converted *SIGLEC11* gene was fixed in human populations. Since *SIGLEC11* and *SIGLEC16* loci are located close together in the genome, this fixation might have driven the high frequency of *SIGLEC16P* alleles in human populations. These are intriguing hypotheses, but need to be examined by further studies.

Siglec-11 and Siglec-16 have coevolved via gene conversions in the human lineage. However, the evolutionary role of observed human-specific changes of Siglec-11 and Siglec-16 is still unknown. To gain deeper insight into the evolutionary relationship between Siglec-11 and Siglec-16, we analyzed the sequences of the *SIGLEC11* and *SIGLEC16* genes not only from human but also from nonhuman primates. We also examined the glycan binding properties of Siglec-11 and Siglec-16 to determine the functional role of gene conversion between Siglec-11 and Siglec-16 because the converted region includes the exon encoding the sialic acid binding domain (i.e., V-set domain).

## Results

### Gene conversions between *SIGLEC11* and *SIGLEC16* in each primate lineage

**Fig. 1:**
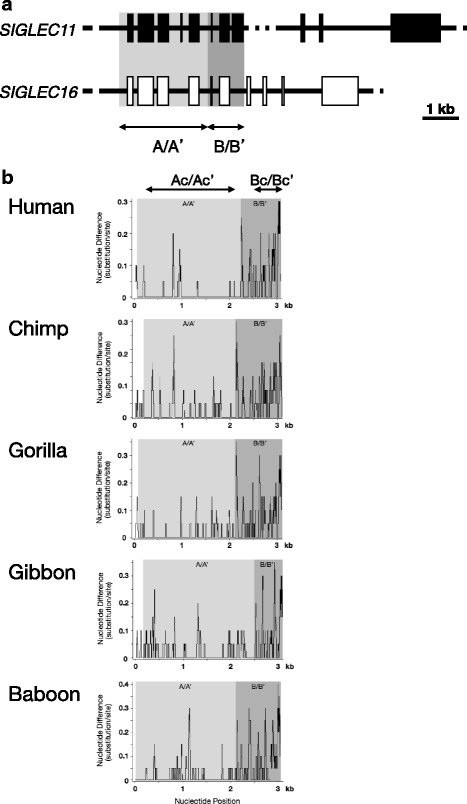
Comparison of the *SIGLEC11* and *SIGLEC16* genes. **a** Gene structures of *SIGLEC11* and *SIGLEC16*. Exons of *SIGLEC11* and *SIGLEC16* are represented by solid and open boxes, respectively. The regions with similarity between *SIGLEC11* and *SIGLEC16* are indicated with a light shadow (A/A’) and a dark shadow (B/B’). **b** Sliding window analysis of the conservation profile of *SIGLEC11* versus *SIGLEC16* (window size 20 bp; step size 1 bp). The ~2-kb genomic region shows consistently high sequence identity (97%–99%) between *SIGLEC11* and *SIGLEC16* in every species, and is designated the Ac/Ac’ region. On the other hand, the ~400-bp genomic region shows consistently lower identity in the similar region of every species, and is designated the Bc/Bc’ region

The genomic region including the first eight exons and a region upstream of the gene is similar between the human *SIGLEC11* and *SIGLEC16* genes (Fig. 1). A (GAAT)n tract is found at the 5′ boundary of these similar regions, and the 3′ boundary is assumed to be around the 3′ end of exon 8 (see Additional file 1: Figure S1). In the human lineage, gene conversions between *SIGLEC11* and *SIGLEC16* occurred in a ~2-kb part of this region, which contains a region upstream of the gene and the exon encoding the sialic acid binding domain (V-set domain) [10, 11]. To determine whether gene conversions have occurred in nonhuman primates, we examined the genomic sequences of chimpanzee, gorilla, gibbon, and baboon. Apes and baboon have both *SIGLEC11* and *SIGLEC16* genes. On the other hand, only the *SIGLEC11* gene is identified in the public databases of the marmoset genome and squirrel monkey genome (data not shown). Thus, it seems that *SIGLEC16* emerged from *SIGLEC11* by gene duplication before the emergence of the common ancestor of Old World monkeys and hominoids. The (GAAT)n tract located at the 5′ boundary of the similar regions is conserved in all species examined, and was thus probably involved in this duplication event. On the other hand, there is no defining sequence motif at the 3′ boundary in all species examined. A frameshift mutation and a large deletion are found in baboon *SIGLEC11* and *SIGLEC16*, respectively (Additional file 1: Figure S1).

A sliding window plot (Fig. 1b) illustrates that the genomic region containing a region upstream of the gene and the first five exons (region A and A’ in *SIGLEC11* and *SIGLEC16*, respectively) shows high sequence identity between *SIGLEC11* and *SIGLEC16* genes in each species: 99.3% in the human, 98.2% in the chimpanzee, 98.5% in the gorilla, 97.8% in the gibbon, and 96.9% in the baboon. The rest of the similar regions (designated B and B’ in *SIGLEC11* and *SIGLEC16*, respectively) show lower identity in all species (93.1% in the human, 93.4% in the chimpanzee, 93.0% in the gorilla, 92.7% in the gibbon, and 87.6% in the baboon). Estimated upstream and downstream boundaries of A/A’ regions differ among the species, and there is no sequence motif signifying these boundaries (see Fig. 1b and Additional file 1: Figure S1).

**Fig. 2:**
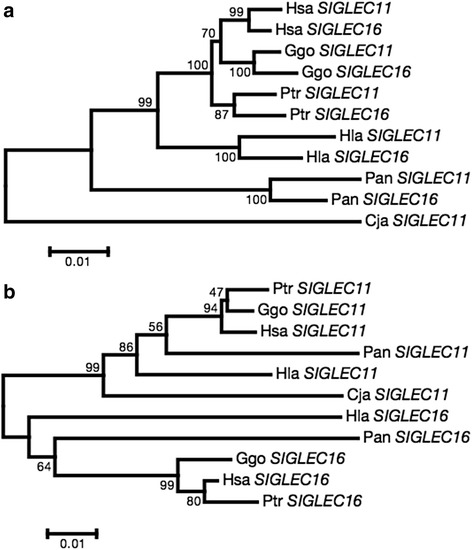
Phylogenetic relationships of (**a**) the Ac/Ac’ region and (**b**) the Bc/Bc’ region of *SIGLEC11* and *SIGLEC16*. Numbers on the phylogenetic tree represent bootstrap values based on 1000 replications. Hsa, *Homo sapiens*; Ptr, *Pan troglodytes*; Ggo, *Gorilla gorilla*; Hla, *Hylobates lar*; Pan, *Papio anubis*; Cja, *Callithrix jacchus*

The ~2-kb genomic region (designated Ac and Ac’ in *SIGLEC11* and *SIGLEC16*, respectively) in the A/A’ region shows high sequence identity (97%–99%) consistently in every species (Fig. 1b). As shown in Fig. 2a, a phylogenetic tree of the Ac/Ac’ region shows that the *SIGLEC11* and *SIGLEC16* genes in each species form a cluster, but do not with their respective orthologs in other species. On the other hand, the ~400-bp genomic region (designated Bc and Bc’ in *SIGLEC11* and *SIGLEC16*, respectively) in the B/B’ region contains the 3′ part of exon 7 and most of exon 8 and shows lower identity consistently in every species (Fig. 1b). The topology of the phylogenetic tree constructed from the Bc/Bc’ region matches expectations given the paralogous relationship between the *SIGLEC11* and *SIGLEC16* genes (Fig. 2b). These phylogenetic trees suggest that gene conversions occurred in the A/A’ regions of every primate species.

It is worth noting that the current sequence comparison among primate *SIGLEC11* and *SIGLEC16* provides further evidence that the GATA-1-binding sequence, which is assumed to be involved in human-specific brain expression of Siglec-11 [10], is modified only in the human genes (see Additional file 1: Figure S1). This strengthens the speculation that the *SIGLEC16P*→*SIGLEC11* gene conversion resulted in the gain of human-specific brain expression of Siglec-11.

### Timing of gene conversions in each primate

We calculated the timing of the gene conversions in the nonhuman primates using noncoding parts of the Ac/Ac’ region. Relative rate tests were performed based on the tree of noncoding parts of the Ac/Ac’ region (Additional file 2: Figure S2), and showed that a rate constancy can be assumed between *SIGLEC11* and *SIGLEC16* genes in chimpanzee, gorilla, and gibbon lineages. The timing (T) of gene conversion can be roughly estimated by T = d/2λ, where d is the genetic distance between the *SIGLEC11* and *SIGLEC16* genes of each species and λ is the neutral mutation rate of the genomic region containing the *SIGLEC11* locus [(1.4 ± 0.1) × 10^-9^/site/year; [10]]. We estimated the timing of most recent gene conversions in chimpanzee, gorilla, and gibbon lineages as 4.4 ± 1.5 MYR, 6.9 ± 1.9 MYR, and 8.4 ± 2.0 MYR, respectively. The preliminary phylogenetic analysis using the partial sequence of bonobo shows that gene conversions in the lineage of the genus *Pan* occurred before the divergence of chimpanzee and bonobo (see Additional file 2: Figure S2). Since chimpanzee and bonobo diverged ~2 MYA [14], the calculated timing (4.4 ± 1.5 MYR) of gene conversion in the *Pan* lineage is consistent with the tree topology shown in Figure S2. The noncoding part (~800 bp) from the Ac/Ac’ region is short, and the current calculation cannot avoid the ambiguity resulting from the use of a very short noncoding part. However, the obtained timing results should be helpful to consider the frequency of gene conversions in the nonhuman primate lineage, and suggests that the frequency of gene conversion is not so high in each nonhuman primate lineage.

### Glycan binding properties of Siglec-11 and Siglec-16

**Fig. 3:**
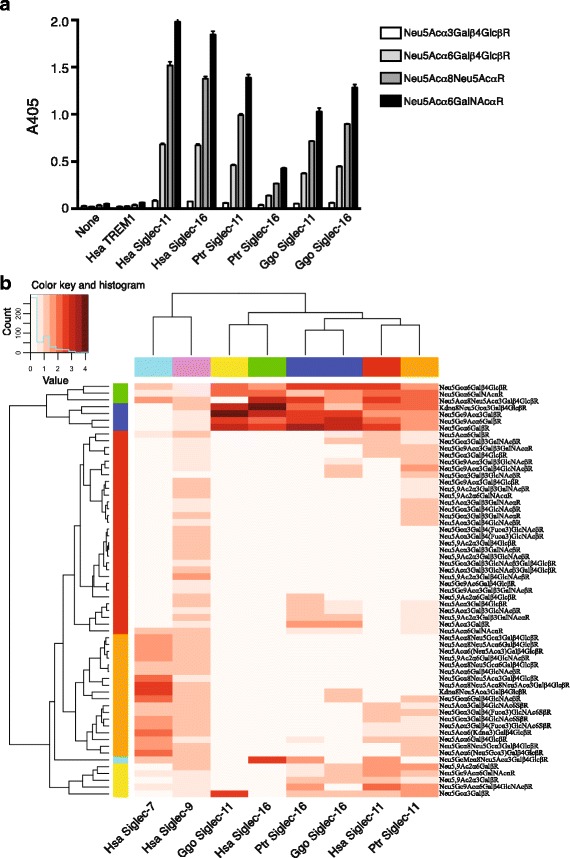
Binding of glycan probes by recombinant Siglec-11 and Siglec-16 from human, chimpanzee, and gorilla. Glycan binding assay was carried out as described in Materials and Methods. **a** Binding of synthetic glycan-polymer probes by Siglecs on solid phase. All recombinant Siglec-Fc proteins tested in the assay showed similar glycan binding preferences, in that the signal intensity of glycan probe binding was as follows: Neu5Acα2-6GalNAc > Neu5Acα2-8Neu5Ac > Neu5Acα2-6Galβ1-4Glc > Neu5Acα2-3Galβ1-4Glc. Wells coated with human TREM-1 (*TREM1*) or without any recombinant protein (*None*) showed negligible binding to these glycan probes and thus were appropriate as negative controls. Binding assays were carried out in triplicate wells for each combination of protein and probe. The experiment was repeated twice with consistent results, and a representative result is shown. Error bar represents standard error of the mean. **b** Binding of Siglecs to glycan microarray. Binding signal intensities were normalized by the root mean square method, and are represented by a heat map (higher color intensity = strong binding). Human Siglec-7 and Siglec-9 were included in the analysis as outgroups. Two different concentrations (20 or 40 μg/ml) of each Siglec-Fc were used in the binding analysis, and gave consistent binding patterns. Trees generated by unsupervised hierarchical clustering of Siglecs (top) and glycans (left), and the glycan structures (right) are shown beside the heat map. Siglec-11 and Siglec-16 from three species are clustered together

The A/A’ region contains the exon encoding the sialic acid binding domain (see Additional file 3: Figure S3). It is thus expected that sequence homogenization by gene conversions has maintained similar glycan binding properties between Siglec-11 and Siglec-16. To test this hypothesis, we performed glycan binding assays using soluble recombinant proteins that include the V-set domain and the adjacent C2-set domain of Siglecs linked to the Fc portion of human immunoglobulin G (Siglec-Fc). We prepared recombinant human, chimpanzee, and gorilla Siglec-11-Fc and Siglec-16-Fc proteins, and tested their binding to glycan-polymer probes. Binding preferences (based on the order of binding signal intensity to the probes) were very similar between Siglec-11 and Siglec-16 in each species as expected, but were also largely conserved across human and great ape species (Fig. 3a). We also used a glycan microarray to analyze ligand specificity in greater detail, and tested whether Siglec-11 and Siglec-16 of the same species show more similar binding patterns compared with their respective orthologs in other species (Fig. 3b). Siglec-11 and Siglec-16 from the three species showed similar binding patterns, distinct from Siglec-7 and Siglec-9. However, the branch topology of the subtree for Siglec-11 and Siglec-16 did not match that of the molecular phylogenetic tree based on the DNA sequences (see Fig. 2a). Taken together, it is reasonable to think that sequence homogenization by gene conversion has maintained similarity in the glycan binding preference between Siglec-11 and Siglec-16 along the primate lineage, but not a strict identity in the glycan binding preference between them in each species.

### Relaxed evolution of Siglec-11/Siglec-16 paired receptors

To gain more insight into the evolutionary background of frequent gene conversion between *SIGLEC11* and *SIGLEC16*, we examined functional constraints on the exons of genomic regions (Ac/Ac’ region) that underwent gene conversion in every primate species by the comparison of branch length between the tree of neutral sites (introns and synonymous sites in exons) and that of nonsynonymous sites. A significant difference between nonsynonymous and neutral substitutions per site is found in only three branches: first, the branch leading to the gorilla *SIGLEC11* gene; second, the branch leading to baboon *SIGLEC11*; and third, the branch leading to baboon genes (Additional file 4: Figure S4). Our analysis has a limitation due to the use of short region (~2 kb), but suggests the relaxation of functional constraints on the exons of the Ac/Ac’ regions of both *SIGLEC11* and *SIGLEC16* genes in the Hominoidea lineage. It is also possible that this relaxed functional constraint might be related to evolutionary acceptance of repeated gene conversions in the A/A’ region, contributing to the maintenance of Siglec-11 and Siglec-16 as paired receptors.

### Dimorphism of Siglec-11/Siglec-16 paired receptors in humans

Immune cell receptors that show significant homology within their extracellular domains and overlapping expression patterns, but opposite roles in signal transduction, are called paired receptors. They are proposed to be involved in the fine-tuning of immune responses via a balance in the ligand binding between the activating and inhibitory members of the pair [15]. Siglec-11 and Siglec-16 are inhibitory and activating receptors, respectively, and expressed in the same cells such as macrophages [8, 9]. Moreover, the glycan binding preferences are very similar between Siglec-11 and Siglec-16, as demonstrated above (Fig. 3). Therefore, Siglec-11 and Siglec-16 are regarded as paired receptors.

Since human *SIGLEC16P* alleles have a 4 base pair deletion in exon 2 and produce a truncated protein lacking a transmembrane domain, human Siglec-11 completely lost its partner as a paired receptor in individuals having only *SIGLEC16P* alleles. We previously reported that the frequency of *SIGLEC16P* is high in every human population [10]. We also updated the frequency of *SIGLEC16P* alleles using recent data from the 1000 Genomes Project (http://browser.1000genomes.org/index.html) (Additional file 5: Figure S5), based on the allele frequencies of SNPs (rs66855472, rs16981577, rs35248548, rs12975261, rs12975940, rs12978378, rs12978610, rs12609492, rs12609509, rs12611411, rs12609811, rs12984584, rs34038717, rs12971775, and rs12985533) that were in perfect linkage disequilibrium with *SIGLEC16*/*SIGLEC16P* polymorphism in the haplotypes identified in our previous work (see Fig. 7c in Wang et al. [10]). These SNPs show the complete agreement in the minor allele frequency with each other in all 26 human populations from the 1000 genome project with the exception of seven SNPs showing unique frequencies in several populations (four populations at rs66855472, two populations at rs12978378, and one population at each rs12978610, rs12611411, rs12609811, rs12971775, and rs12985533) (Additional file 6: Table S1). Since the number of individuals in each population is the same between the SNPs, the discrepancy in frequencies in several populations at the seven SNPs is likely due to recombination or genotyping error. Data for all of these SNP sites indicate that the frequency of *SIGLEC16P* alleles (65%–91%) is much higher than that of *SIGLEC16* alleles in every human population (Additional file 5: Figure S5 and Additional file 6: Table S1). In modern human populations, over a half of individuals have only *SIGLEC16P* alleles.

## Discussion

Our study demonstrated that, in addition to the *SIGLEC16P*→*SIGLEC11* gene conversion in the human lineage [10, 11], the gene conversion between *SIGLEC11* and *SIGLEC16* has occurred in each nonhuman primate lineage. The region converted in common in all primate species contains first five exons of *SIGLEC11* and first four exons of *SIGLEC16* (see Fig. 1b), and is smaller than the region that was used to detect the *SIGLEC16P*→*SIGLEC11* gene conversion by the comparison between the human and chimpanzee sequences [10, 11]. These exons of the two *SIGLEC* genes have undergone repeated gene conversion along the primate lineage. Sequence homogenization by gene conversion appears to have maintained the glycan binding preferences of Siglec-11/Siglec-16 paired receptors similar. It is thus possible to conclude that sequence homogenization between first five exons of *SIGLEC11* and first four exons of *SIGLEC16* are essential to maintain Siglec-11 and Siglec-16 as paired receptors in the primate lineage. The frequency of gene conversions is inversely proportional to the distance between the interacting sequences in cis [16]. The A region of *SIGLEC11* gene is located about 9 kb apart from A’ of *SIGLEC16* gene. This proximity of genes seems to be an important factor that has caused frequent gene conversions in the evolution of Siglec-11/Siglec-16 paired receptors.

The sliding window analysis indicates that actual converted regions are fragmented in the A/A’ region of each primate (Fig. 1b). In addition, the gene conversion occurred ~1 MYA in the human lineage [10], and the gene conversion have not occurred during at least 2 MYR in the *Pan* lineage. These suggest that some degree of sequence differences between the *SIGLEC11* and *SIGLEC16* genes has been accepted in each species. This is consistent with our observation that Siglec-11 and Siglec-16 did not show a strict identity in the glycan binding preferences (see Fig. 3), and thus may imply that such strict identity is not essential for them to function as paired receptors.

Some pathogens engage inhibitory Siglecs, and attenuate immune responses. We proposed activating Siglecs may have emerged as a counter-measure against pathogens that exploit inhibitory counterparts [17]. The Siglec-11/Siglec-16 paired receptors might have emerged and have been maintained under the interaction with pathogens that target macrophage (see below) along the primate lineage. The relaxation of functional constraints was deduced on the exons of the Ac/Ac’ regions of both *SIGLEC11* and *SIGLEC16* genes in the Catarrhini lineage. This relaxation of functional constraint might have contributed to an arms race against pathogens, by allowing a rapid functional evolution of these Siglec proteins. Interestingly, in addition to human Siglec-11 and Siglec-16 for which expression in the brain emerged, chimpanzee and gorilla Siglecs-11 and -16 show binding to Neu5Acα2-8Neu5Ac (α2-8-linked sialic acids), a glycan structure that is enriched in the brain (Fig. 3). The relaxation of functional constraint might have contributed to the acquisition of binding to α2-8-linked sialic acids before Siglec-11 and Siglec-16 started to be expressed in the brain in the human lineage, namely, exaptation, for binding to brain sialic acids, and enabled Siglec-11 and Siglec-16 to participate as functional receptors in brain immunity.

If Siglec-16 is functionally essential for human biology, the *SIGLEC16P* allele should be deleterious in human evolution. The possible functional importance of Siglec-16 in human immunity has been suggested by our recent study demonstrating its interaction with the human-specific pathogen *Escherichia coli* K1 that produces a capsular polysaccharide made of α2-8-linked sialic acids, which is a perfect mimic of the preferred ligand of Siglec-11/Siglec-16 paired receptors [18]. It is therefore reasonable to assume that Siglec-16 is involved in the host defense against some bacteria. However, the *SIGLEC16P* allele has persisted for over 3 MYR, and shows very high frequency in human populations. The presence of functional Siglec-16 protein may be deleterious under certain circumstances while being beneficial under others, as is the case for Siglec-14 [19, 20].

Only humans have been shown to have both Siglec-11 and Siglec-16 as paired receptors in the brain microglia. Human Siglec-11 came to be expressed in the brain microglia even after the *SIGLEC16P* allele had emerged, which indicates that the human had Siglec-16 and Siglec-16P in brain microglia before Siglec-11 gained brain expression. Siglec-11 shows neuroprotective effects such as inhibition of the production of proinflammatory mediators, and likely plays an important role as an inhibitory receptor in the immune function of microglia [13]. Siglec-16 activates immune and inflammatory responses in the brain microglia, and the activation of brain microglia is associated with mental disorders such as schizophrenia [21]. It is thus tempting to speculate that the partial elimination of Siglec-16 already occurred to reduce the unnecessary activation of brain microglia before the gain of Siglec-11 in the brain microglia, and Siglec-11 then came to be expressed in the brain microglia, which led to the further suppression of this unnecessary activation even in the individual who has Siglec-16. Siglec-11 might have contributed to the human brain evolution by its neuroprotective effect via inhibition of unnecessary activation of microglia. Further investigation would be needed to examine the evolutionary role of dimorphism of Siglec-11/Siglec-16 paired receptors.

## Conclusions

Siglec-11 and Siglec-16 are inhibitory and activating receptors, respectively, and expressed in the same cells such as macrophages [8, 9]. Similar glycan binding preferences between Siglec-11 and Siglec-16 have been maintained by sequence homogenization via gene conversion in each primate lineage. Thus, Siglec-11 and Siglec-16 are regarded as paired receptors, and repeated gene conversions have played an important role in the evolution of Siglec-11/Siglec-16 paired receptors in the primate lineage. The relaxed functional constraint on the exons of the converted regions of both *SIGLEC11* and *SIGLEC16* genes seems to have contributed to the maintenance of Siglec-11 and Siglec-16 as paired receptors by the acceptance of repeated gene conversions. The high prevalence of the nonfunctional *SIGLEC16P* allele and the fixation of the converted *SIGLEC11* in human populations imply that the dimorphism of Siglec-11/Siglec-16 paired receptors, namely, Siglec-11/Siglec-16 and Siglec-11/Siglec-16P, have been maintained under some evolutionary constraint in the human lineage.

## Methods

### Sequences of *SIGLEC11* and *SIGLEC16* genes

The human *SIGLEC11* and *SIGLEC16* genes show sequence similarity in the ~3-kb region containing a region upstream of the gene and the first eight exons [8, 10, 11]. We targeted these similar genomic regions (target regions) in this study. In the public databases, the complete genomic sequences of target regions of both the *SIGLEC11* gene and the *SIGLEC16* gene are available from the human (*Homo sapiens*), the chimpanzee (*Pan troglodytes*), and the baboon (*Papio anubis*). However, the target regions of the *SIGLEC16* gene are incomplete in the public databases of other ape genomes. We thus obtained the complete sequences from the gorilla (*Gorilla gorilla*) and the gibbon (*Hylobates lar*) by using genomic PCR (GenBank accession nos. LC194903–194906). In addition to these two primates, human and chimpanzee sequences were also amplified for the construction of expression vectors of glycan binding assays (see the section on the preparation of recombinant Siglec fusion protein). Human genomic DNA was purchased at Coriell Cell Repositories, and ape genomic DNA samples were provided from Max Planck Institute for Biology. The PCR primers (HS11F0, HS11R0, HS11R0G, HS16F1, HS16R2, and HS16R2G; see Additional file 7: Table S2) were designed based on the genomic sequences of the human, chimpanzee, and gorilla in the public database (http://www.ncbi.nlm.nih.gov). PCR reactions were performed with 20 pmol of each primer and 2–5 μl of the genomic DNA solution in a total volume of 50 μl containing 200 μM dNTPs and 2.5 units of EX-Taq DNA polymerase (Takara, Otsu, Japan) in PCR buffer containing 2 mM MgCl_2_. The PCR conditions were as follows: denaturation at 95°C for 5 min; followed by 40 cycles of 95°C for 1 min, 60–68°C for 1 min, and 72°C for 5 min; and final extension at 72°C for 10 min. As for the gorilla, nested PCR using primers (GS11F3, GS11R3, GS16F2, and GS16R2; see Additional file 7: Table S2) was performed to obtain a sufficient amount of PCR products for sequencing and vector construction for the glycan binding assay. The conditions of nested PCR were as follows: denaturation at 95°C for 5 min; followed by 35 cycles of 95°C for 1 min, 62°C for 1 min, and 72°C for 5 min; and final extension at 72°C for 10 min. The obtained PCR products were purified using the QIAquick PCR Purification Kit (Qiagen) and directly sequenced on an ABI 3130 genetic analyzer (Applied Biosystems, Foster City, CA).

Sequences of target regions of baboon *SIGLEC11* and *SIGLEC16* genes were obtained from the baboon genome sequences (Baylor Panu_2.0/papAnu2; UCSC Genome Browser, http://genome.ucsc.edu/cgi-bin/hgGateway). We also obtained the sequence of the *SIGLEC11* gene from the marmoset (*Callithrix jacchus*) genome database (WUGSC 3.2/caljac3; UCSC Genome Browser, http://genome.ucsc.edu/cgi-bin/hgGateway).

### Sequence analysis

DNASIS software (Hitachi, Tokyo, Japan) was used to assemble sequences. Phylogenetic tree construction was performed by the neighbor-joining method with multiple-hit corrections using MEGA5 software [22–24]. DnaSP version 3 [25] was used to obtain a sliding window plot representing the nucleotide differences.

To examine functional constraints on the exons in genomic regions that underwent gene conversion, lineage-specific ratios of nonsynonymous substitutions per site and neutral substitutions per site were examined. In this analysis, intronic sites and synonymous sites were used to estimate neutral substitution. A phylogenetic tree of introns was constructed, and its tree topology was used as a reference. Based on this reference tree topology, the number of substitutions of every branch was estimated for synonymous sites and nonsynonymous sites, separately, by using the least squares method. Neutral substitutions per site for each lineage were obtained from the average of synonymous substitutions per site and intronic substitutions per site. Z-tests were performed to examine the significance of differences in lineage-specific ratios of nonsynonymous substitutions per site to neutral substitutions per site.

### Preparation of recombinant Siglec-11-Fc and Siglec-16-Fc fusion proteins

Recombinant proteins, consisting of the “immunoglobulin-like domains 1 + 2” of Siglec-11 or Siglec-16 of human, chimpanzee, and gorilla and the “hinge + Fc” region of human IgG, were prepared as follows.

The genomic DNA segments encompassing exons 1 through 4 of the *SIGLEC11* gene or the *SIGLEC16* gene of respective species (approx. 1.3 kb) were amplified by using appropriate genomic DNA templates and primer pairs (Additional file 8: Table S3), using Phusion High-Fidelity DNA Polymerase (Finnzymes/Thermo Scientific). Amplified DNA fragments were digested with Xba I (New England Biolabs) and cloned into Xba I–EcoRV sites of the AvT-EK-Fc/pcDNA vector [26], and the sequences of the resulting constructs were confirmed. As the yields of recombinant proteins produced by using these constructs were generally poor (data not shown), removal of introns from the Siglec-coding segments was carried out as follows.

The 293T human embryonic kidney cell line was transiently transfected with the constructs prepared as above using LipofectAMINE 2000 (Life Technologies), and the total RNA was prepared from each transfectant using RNeasy Mini Kit (Qiagen). The RNA samples (1 μg each) were reverse-transcribed and subjected to PCR using Phusion DNA polymerase and the same primer pairs as employed for the amplification of respective genomic DNA fragments. The resulting PCR products were purified, subcloned to Xba I–EcoRV sites of the AvT-EK-Fc/pcDNA vector, and the sequences of the resulting constructs were confirmed. These expression constructs were given systematically named using the three-letter abbreviation of the systematic name of the species of origin and the Siglec number (i.e., Hsa Siglec-11 or 16-Fc/pcDNA, Ptr Siglec-11 or 16-Fc/pcDNA, and Ggo Siglec-11 or 16-Fc/pcDNA).

Production and purification of Siglec-Fc fusion proteins were carried out as previously described [17, 27]. In short, 293T cells (or 293A cells) were transiently transfected with the recombinant protein expression constructs using LipofectAMINE 2000. The culture medium was switched to 2% low IgG fetal bovine serum (HyClone/Thermo Scientific) in Opti-MEM (Life Technologies) the next day, and the culture supernatant was collected on third and sixth days after changing medium. Recombinant Siglec-Fc proteins were purified from the combined culture supernatant by adsorption to Protein A Sepharose (GE Healthcare Life Sciences), with on-column treatment with *Arthrobacter ureafaciens* sialidase to remove sialic acids from the Siglec-Fc proteins themselves, and used for binding assays.

### Analysis of binding between synthetic glycan probes and Siglec-Fc

Analysis of the binding between glycan probes and Siglec-Fc was performed as described previously [27], with minor modifications. In brief, soluble protein A (0.5 μg/well; Sigma-Aldrich) was immobilized onto a 96-well plate (#269620, Corning) by overnight incubation in alkaline buffer (50 mM sodium bicarbonate buffer, pH 9.5) at 4°C. In the subsequent steps, incubations were carried out at room temperature (RT), and the wells were washed three times with 1% bovine serum albumin in Dulbecco’s phosphate-buffered saline (blocking buffer) in-between incubations with different reagents. The wells were blocked with blocking buffer for 1 h, and Siglec-Fc proteins (0.5 μg/well) were adsorbed to the wells for 3 h. The wells were further incubated with biotinylated polyacrylamide-based probes carrying multiple copies of synthetic oligosaccharides (glycan probes, 0.5 μg/well; GlycoTech) for 2 h. Each combination of recombinant protein and probe was tested in triplicate wells. The wells were further incubated with streptavidin-conjugated alkaline phosphatase (1 μg/well; Jackson ImmunoResearch) for 2 h, followed by incubation with alkaline phosphatase substrate solution (10 mM *p*-nitrophenyl phosphate, 1 mM MgCl_2_, 100 mM Na_2_CO_3_). Absorption at 405 nm was monitored with a microplate reader (VersaMax, Molecular Devices) at different time points up to 60 min.

### Analysis of Siglec-Fc binding to glycan microarray

Glycan microarrays were fabricated as described previously (array 1; [28]). Each glycan was represented by four spots per subarray in 100 μM in an optimized print buffer (300 mM phosphate buffer, pH 8.4). Printed glycan microarray slides were blocked by ethanolamine, washed, and dried. Slides were then fitted in a multi-well microarray hybridization cassette (AHC4X8S; ArrayIt, Sunnyvale, CA, USA) for division into eight subarrays (each subarray comprising the complete set of glycans). The subarrays were blocked with ovalbumin (1% w/v) in PBS (pH 7.4) for 1 h at RT, with gentle shaking. Subsequently, the blocking solution was removed and Siglec-Fc (400 μl, at 20 or 40 μg/ml) was applied to each subarray. After incubating the samples for 2 h at RT with gentle shaking, the slides were washed. Diluted goat anti-human IgG-Cy3 antibody (Jackson ImmunoResearch Laboratories) in PBS was added to the subarrays, incubated for 1 h at RT, washed, and dried. The microarray slides were scanned by a Genepix 4000B microarray scanner (Molecular Devices Corp., Union City, CA, USA). Data analysis was performed using Genepix Pro 7.0 analysis software (Molecular Devices Corp.), and Siglec-Fc binding to each glycan was quantified as the relative fluorescence intensity (RFU) of spots by subtraction of the background and averaging of four spots for the same glycan.

### Cluster analysis of glycan microarray data

Cluster analysis of glycan microarray data was conducted by using R (version 3.1.1, https://www.R-project.org), with add-on packages. Binding data in RFU were normalized by the root mean square method. The unsupervised hierarchical clustering of Siglecs and glycans was performed by a complete linkage method with the hclust function, in which a dissimilarity structure was calculated by using Euclidean distance.

## Additional files

Additional file 1: Figure S1. Alignment of genomic nucleotide sequences of *SIGLEC11* and *SIGLEC16* in human, chimpanzee, gorilla, gibbon, baboon, and marmoset. The genomic region of human *SIGLEC11* including the first eight exons is aligned with the corresponding regions of *SIGLEC11* and *SIGLEC16* from other primates. Dots indicate nucleotides identical to those of human *SIGLEC11*. Dashes indicate gaps used for sequence alignment. Exons are marked with the red bars lying on the sequences. Red open box indicates the ATG start codon. The arrows indicate the boundaries of A/A’ and B/B’ regions (see also Figure 1B). The putative GATA-1-binding sequence is underlined. The *SIGLEC16P* sequence is represented as human sequence of *SIGLEC16* gene because of the *SIGLEC16P*→*SIGLEC11* gene conversion in the human lineage [10, 11]. Hsa, *Homo sapiens*; Ptr, *Pan troglodytes*; Ggo, *Gorilla gorilla*; Hla, *Hylobates lar*; Pan, *Papio anubis*; Cja, *Callithrix jacchus*. (PDF 2906 kb)

Additional file 2: Figure S2. Phylogenetic relationships of the A/A’ regions of *SIGLEC11* and *SIGLEC16*. The partial sequence of bonobo *SIGLEC11* was obtained previously (GenBank accession no. AB211392; [11]) and used in the tree construction. The tree topology is identical to that shown in Fig. 2A, with the exception of genes of the genus *Pan*. As for the lineage of the genus *Pan*, bonobo *SIGLEC11* is most closely related to chimpanzee *SIGLEC11* but the genes of genus *Pan* form a cluster in the tree. This suggests that gene conversion between *SIGLEC11* and *SIGLEC16* occurred before the divergence of chimpanzee and bonobo in the lineage of the genus *Pan*. Numbers on the phylogenetic tree represent bootstrap values based on 1000 replications. Hsa, *Homo sapiens*; Ptr, *Pan troglodytes*; Ppa, *Pan paniscus*; Ggo, *Gorilla gorilla*; Hla, *Hylobates lar*; Pan, *Papio anubis*; Cja, *Callithrix jacchus*. (PDF 45 kb)

Additional file 3: Figure S3. Alignment of amino acid sequences of Siglec-11 and Siglec-16 proteins in primates. Amino acid sequences of primate Siglec-11 and Siglec-16 (signal peptide + first and second immunoglobulin-like domains) were aligned by ClustalO. The amino acid position fully conserved among all aligned Siglec-11 and Siglec-16 is marked with asterisk (*), and the position conserved within groups of strongly or weakly similar amino acids (based on the properties of side chain) is marked with colon (:) or period (.), respectively. Functional human Siglec-16 sequence was used for the alignment. Hsa: *Homo sapiens*; Ptr: *Pan troglodytes*; Ggo: *Gorilla gorilla*; Hla: *Hylobates lar*; Pan: *Papio anubis*; Cja: *Callithrix jacchus*. Residues important for sialic acid recognition are marked with colored squares. Because the atomic level structure of Siglec-11/-16 is not available at present, amino acid residues known to be important for glycan recognition by Siglecs in common (based on the atomic level structures of Siglec-1, -2, -4, -5, and -7 in complex with respective ligand) are indicated. Red square: essential arginine residue interacting with the carboxyl group of sialic acid; Orange squares: aromatic amino acid residues involved in the coordination of sialic acid. (PDF 36 kb)

Additional file 4: Figure S4. Relaxed evolution of exons that underwent gene conversions. The phylogenetic tree of introns was used as a reference to examine functional constraints. The topology of the intron tree is similar to that of the Ac/Ac’ region, representing that gene conversion occurred in each primate lineage (Figure 2A). Lineage-specific ratios of nonsynonymous substitutions per site to silent substitutions per site (at both synonymous and intron sites) are shown on each branch. A significant difference between nonsynonymous substitutions per site and neutral substitutions per site is found in only three branches, one leading to gorilla *SIGLEC11*, one leading to baboon *SIGLEC11*, and the other leading to two baboon genes (P<0.02, Z-test). These branches are represented by bold lines. Hsa, *Homo sapiens*; Ptr, *Pan troglodytes*; Ggo, *Gorilla gorilla*; Hla, *Hylobates lar*; Pan, *Papio anubis*; Cja, *Callithrix jacchus*. (PDF 40 kb)

Additional file 5: Figure S5. Distribution of *SIGLEC16* and *SIGLEC16P* alleles in human populations. Pie charts represent the proportion of each allele type by geographic regions. The frequencies at rs12984584 are used as a representative in this figure. 1, Americans of African ancestry (USA); 2, Gambian in Western Divisions (The Gambia); 3, Mende (Sierra Leone); 4, Esan (Nigeria); 5, Yoruba in Ibadan (Nigeria); 6, Luhya in Webuye (Kenya); 7, Utah residents (CEPH) with Northern and Western European ancestry; 8, Finnish (Finland); 9, British in England and Scotland; 10, Iberian population (Spain); 11, Tuscans (Italy); 12, Punjabi from Lahore (Pakistan); 13, Gujarati Indian from Houston (USA); 14, Indian Telugu from the UK; 15, Sri Lankan Tamil from the UK; 16, Bengali (Bangladesh); 17, Han Chinese in Bejing (China); 18, Southern Han Chinese (China); 19, Chinese Dai in Xishuangbanna (China); 20, Kinh in Ho Chi Minh City (Vietnam); 21, Japanese in Tokyo (Japan); 22, Mexican ancestry from Los Angeles (USA); 23, Puerto Ricans (Puerto Rico); 24, African Caribbeans (Barbados); 25, Colombians from Medellin (Colombia); and 26, Peruvians from Lima (Peru). Original genotyping data were obtained from the website of the 1000 Genomes Project (http://browser.1000genomes.org/index.html). (PDF 463 kb)

Additional file 6: Table S1. Frequencies of *SIGLEC16* and *SIGLEC16P* in human populations. (XLSX 18 kb)

Additional file 7: Table S2. Primer sequences for genomic PCR. (XLSX 9 kb)

Additional file 8: Table S3. Primer sequences for preparation of recombinant proteins. (XLSX 9 kb)

## Abbreviations

ITAM: Immunoreceptor tyrosine-based activating motif
ITIM: Immunoreceptor tyrosine-based inhibitory motif
MYA: Million years ago
MYR: Million years
Siglecs: Sialic acid-binding immunoglobulin superfamily lectins
