# Supplementary material for: Coevolution of Siglec-11 and Siglec-16 via gene conversion in primates
Source: BMC Evol Biol. 2017 Nov 23;17:228. doi: 10.1186/s12862-017-1075-z (PMC5701461; doi:10.1186/s12862-017-1075-z)
Supplement: Supplementary file 1 — Alignment of genomic nucleotide sequences of SIGLEC11 and SIGLEC16 in human, chimpanzee, gorilla, gibbon, baboon, and marmoset. The genomic region of human SIGLEC11 including the first eight exons is aligned with the corresponding regions of SIGLEC11 and SIGLEC16 from other primates. Dots indicate nucleotides identical to those of human SIGLEC11. Dashes indicate gaps used for sequence alignment. Exons are marked with the red bars lying on the sequences. Red open box indicates the ATG start codon. The arrows indicate the boundaries of A/A’ and B/B’ regions (see also Figure 1B). The putative GATA-1-binding sequence is underlined. The SIGLEC16P sequence is represented as human sequence of SIGLEC16 gene because of the SIGLEC16P→SIGLEC11 gene conversion in the human lineage [10, 11]. Hsa, Homo sapiens; Ptr, Pan troglodytes; Ggo, Gorilla gorilla; Hla, Hylobates lar; Pan, Papio anubis; Cja, Callithrix jacchus. (PDF 2906 kb) [file 12862_2017_1075_MOESM1_ESM.pdf]

Figure S1 Hayakawa et al.

```
Hsa SIGLEC11.seq     1 ------------GAATGAATGAATGAATGAGACTCTGCATCCATCACCTCACCTGCAGCC    48
Hsa SIGLEC16.seq     1 --------GAAT.................G.................T............    52
Ptr SIGLEC11.seq     1 ----GAATGAAT.................G.............................T    56
Ptr SIGLEC16.seq     1 ----GAATGAAT................................................    56
Ggo SIGLEC11.seq     1 ----GAATGAAT.................G.............................T    56
Ggo SIGLEC16.seq     1 ----GAATGAAT.................G.........C....................    56
Hla SIGLEC11.seq     1 GAATAAATGAAT..............................T.................    60
Hla SIGLEC16.seq     1 ----GAATGAAT.................G..............................    56
Pan SIGLEC11.seq     1 GAATGAATGAAT.................G..............................    60
Pan SIGLEC16.seq     1 GAATGAATGAAT.................G..............................    60
Cja SIGLEC11.seq     1 --------------------.........G..........T...................    40

Hsa SIGLEC11.seq    49 GTGACTCACCTGTGTCCCCTGACAGTCCCCTCCTAGCACTGCCCACCTCCGAGGCTGGCC   108
Hsa SIGLEC16.seq    53 A...........................................................   112
Ptr SIGLEC11.seq    57 ..................................................A.........   116
Ptr SIGLEC16.seq    57 A...........................................................   116
Ggo SIGLEC11.seq    57 ..............C.........A....................T..............   116
Ggo SIGLEC16.seq    57 A.......................A....................T..............   116
Hla SIGLEC11.seq    61 A...................................................C.......   120
Hla SIGLEC16.seq    57 A........................................................A..   116
Pan SIGLEC11.seq    61 A..............................................C............   120
Pan SIGLEC16.seq    61 A..............................................C............   120
Cja SIGLEC11.seq    41 A.A.........................T......A...........C.........C..   100

Hsa SIGLEC11.seq   109 AGGGTGGCTCCTTCCTGGTTTGGGGCTGGTCCTCATGCCCTCTGCCCTGCTGCTTCCCCT   168
Hsa SIGLEC16.seq   113 ............................................................   172
Ptr SIGLEC11.seq   117 ...........................A................................   176
Ptr SIGLEC16.seq   117 ............................................................   176
Ggo SIGLEC11.seq   117 .......T....................................................   176
Ggo SIGLEC16.seq   117 ............................................................   176
Hla SIGLEC11.seq   121 ............................................................   180
Hla SIGLEC16.seq   117 ............................................................   176
Pan SIGLEC11.seq   121 .............................-..............................   179
Pan SIGLEC16.seq   121 .............................-..............................   179
Cja SIGLEC11.seq   101 ..........................A............................T....   160
```

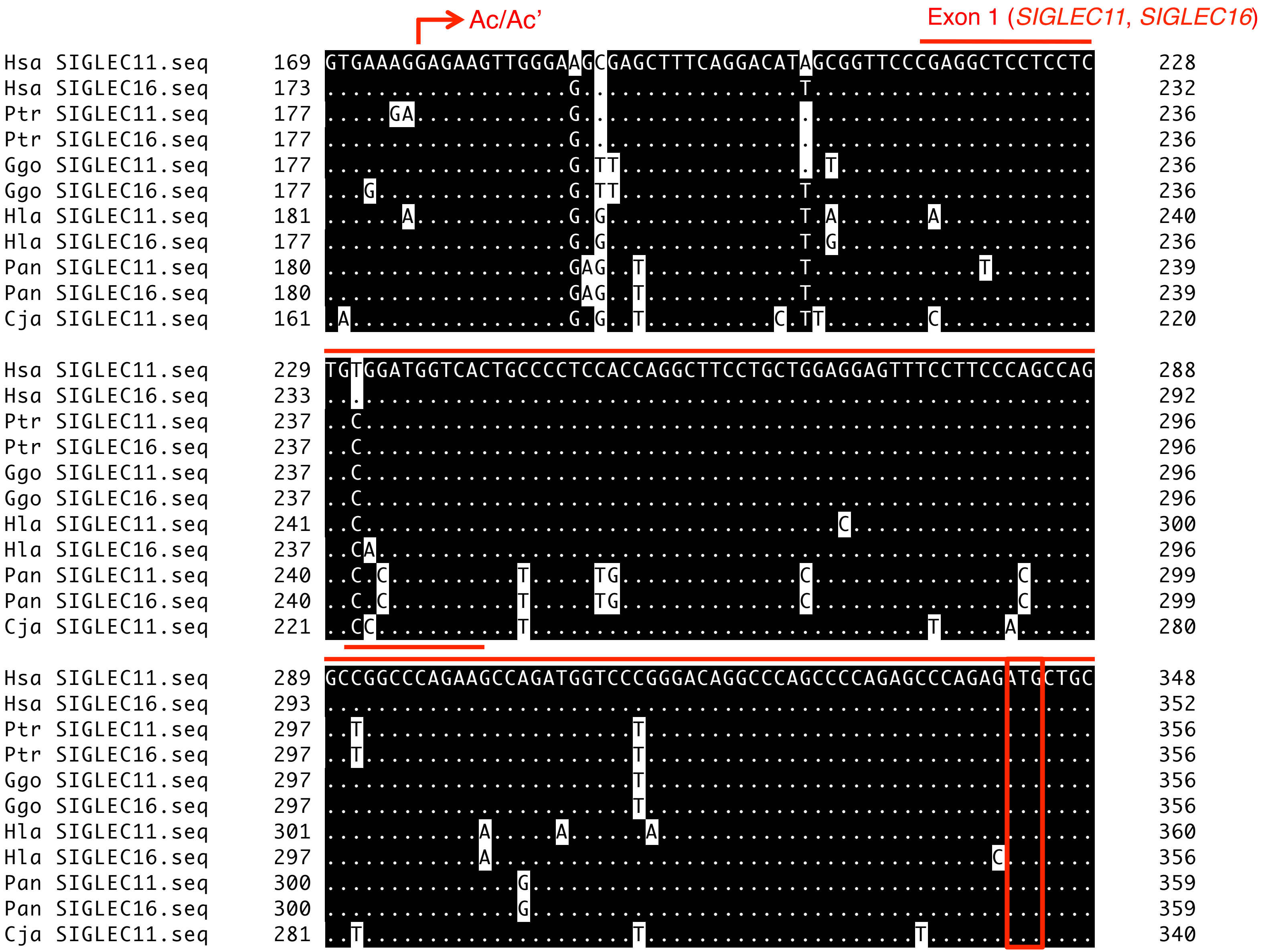
Ac/Ac'
Exon 1 (*SIGLEC11*, *SIGLEC16*)
Hsa SIGLEC11.seq 169 GTGAAAGGAGAAGTTGGGAAGCGAGCTTTCAGGACATAGCGGTTCCCGAGGCTCCTCCTC 228
Hsa SIGLEC16.seq 173 232
Ptr SIGLEC11.seq 177 236
Ptr SIGLEC16.seq 177 236
Ggo SIGLEC11.seq 177 236
Ggo SIGLEC16.seq 177 236
Hla SIGLEC11.seq 181 240
Hla SIGLEC16.seq 177 236
Pan SIGLEC11.seq 180 239
Pan SIGLEC16.seq 180 239
Cja SIGLEC11.seq 161 220
Hsa SIGLEC11.seq 229 TGTGGATGGTCACTGCCCCTCCACCAGGCTTCCTGCTGGAGGAGTTTCCTTCCCAGCCAG 288
Hsa SIGLEC16.seq 233 292
Ptr SIGLEC11.seq 237 296
Ptr SIGLEC16.seq 237 296
Ggo SIGLEC11.seq 237 296
Ggo SIGLEC16.seq 237 296
Hla SIGLEC11.seq 241 300
Hla SIGLEC16.seq 237 296
Pan SIGLEC11.seq 240 299
Pan SIGLEC16.seq 240 299
Cja SIGLEC11.seq 221 280
Hsa SIGLEC11.seq 289 GCCGGCCCAGAAGCCAGATGGTCCCGGGACAGGCCCAGCCCCAGAGCCCAGAGATGCTGC 348
Hsa SIGLEC16.seq 293 352
Ptr SIGLEC11.seq 297 356
Ptr SIGLEC16.seq 297 356
Ggo SIGLEC11.seq 297 356
Ggo SIGLEC16.seq 297 356
Hla SIGLEC11.seq 301 360
Hla SIGLEC16.seq 297 356
Pan SIGLEC11.seq 300 359
Pan SIGLEC16.seq 300 359
Cja SIGLEC11.seq 281 340

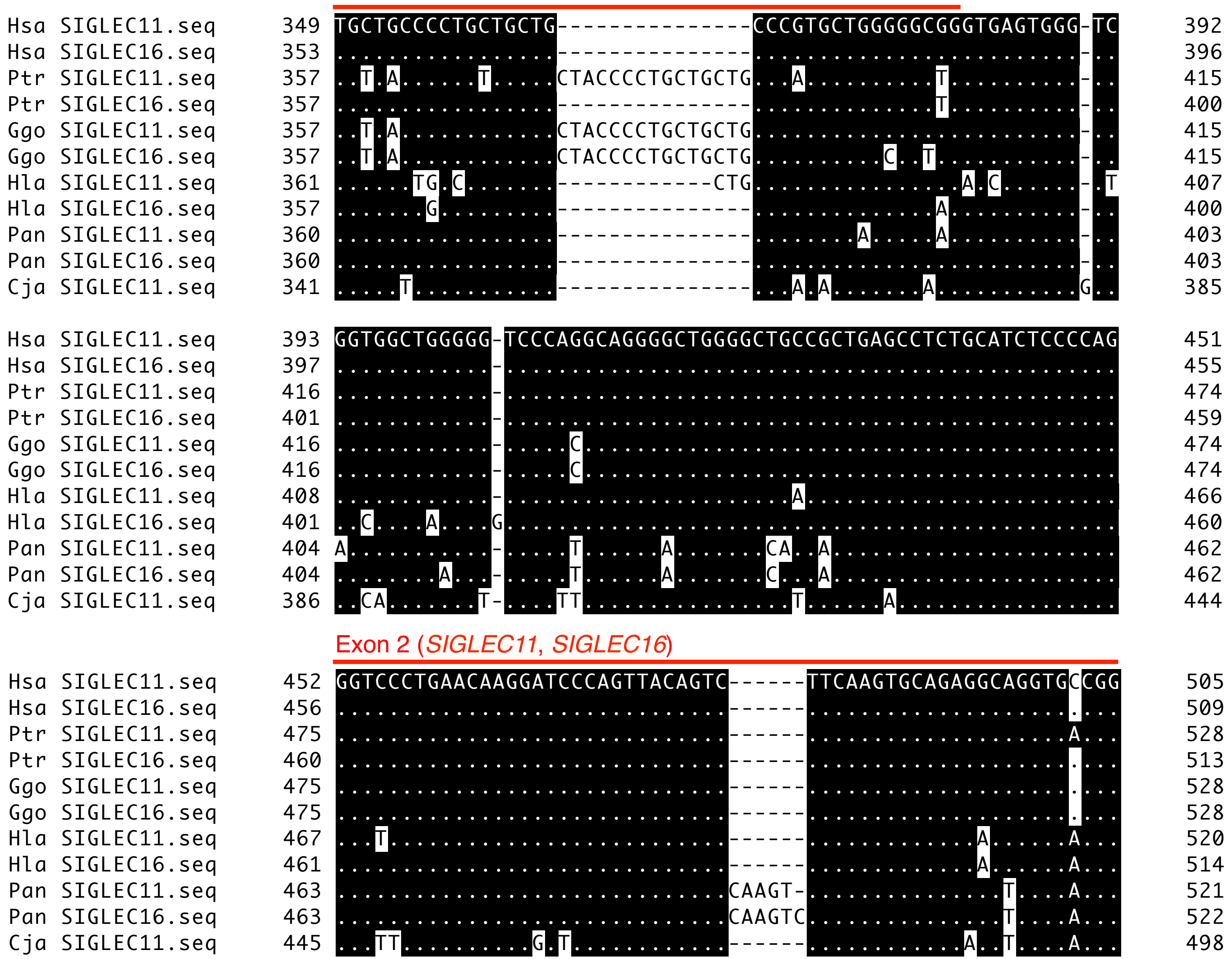
Hsa SIGLEC11.seq 349 TGCTGCCCCTGCTGCTG---------------CCCGTGCTGGGGGCGGGTGAGTGGG-TC 392
Hsa SIGLEC16.seq 353 .................---------------.........................-.. 396
Ptr SIGLEC11.seq 357 ..T.A......T.....CTACCCCTGCTGCTG...A..........T..........-.. 415
Ptr SIGLEC16.seq 357 .................---------------..............T..........-.. 400
Ggo SIGLEC11.seq 357 ..T.A............CTACCCCTGCTGCTG.........................-.. 415
Ggo SIGLEC16.seq 357 ..T.A............CTACCCCTGCTGCTG..........C..T...........-.. 415
Hla SIGLEC11.seq 361 ......TG.C.......------------CTG................A.C......-.T 407
Hla SIGLEC16.seq 357 .......G.........---------------..............A..........-.. 400
Pan SIGLEC11.seq 360 .................---------------........A.....A..........-.. 403
Pan SIGLEC16.seq 360 .................---------------.........................-.. 403
Cja SIGLEC11.seq 341 .....T...........---------------...A.A.......A...........G.. 385
Hsa SIGLEC11.seq 393 GGTGGCTGGGGG-TCCCAGGCAGGGGCTGGGGCTGCCGCTGAGCCTCTGCATCTCCCCAG 451
Hsa SIGLEC16.seq 397 ....-... 455
Ptr SIGLEC11.seq 416 ....-... 474
Ptr SIGLEC16.seq 401 ....-... 459
Ggo SIGLEC11.seq 416 ............-.....C... 474
Ggo SIGLEC16.seq 416 ............-.....C... 474
Hla SIGLEC11.seq 408 ............-......................A........................ 466
Hla SIGLEC16.seq 401 ..C....A...G.. 460
Pan SIGLEC11.seq 404 A...........-.....T......A.......CA..A...................... 462
Pan SIGLEC16.seq 404 ........A...-.....T......A.......C...A...................... 462
Cja SIGLEC11.seq 386 ..CA.......T-....TT................T......A................. 444
Exon 2 (SIGLEC11, SIGLEC16)
Hsa SIGLEC11.seq 452 GGTCCCTGAACAAGGATCCCAGTTACAGTC------TTCAAGTGCAGAGGCAGGTGCCGG 505
Hsa SIGLEC16.seq 456 ....------........................ 509
Ptr SIGLEC11.seq 475 ..............................------....................A... 528
Ptr SIGLEC16.seq 460 ....------........................ 513
Ggo SIGLEC11.seq 475 ....------........................ 528
Ggo SIGLEC16.seq 475 ....------........................ 528
Hla SIGLEC11.seq 467 ...T..........................------.............A......A... 520
Hla SIGLEC16.seq 461 ..............................------.............A......A... 514
Pan SIGLEC11.seq 463 ..............................CAAGT-...............T....A... 521
Pan SIGLEC16.seq 463 ..............................CAAGTC...............T....A... 522
Cja SIGLEC11.seq 445 ...TT..........G.T............------............A..T....A... 498

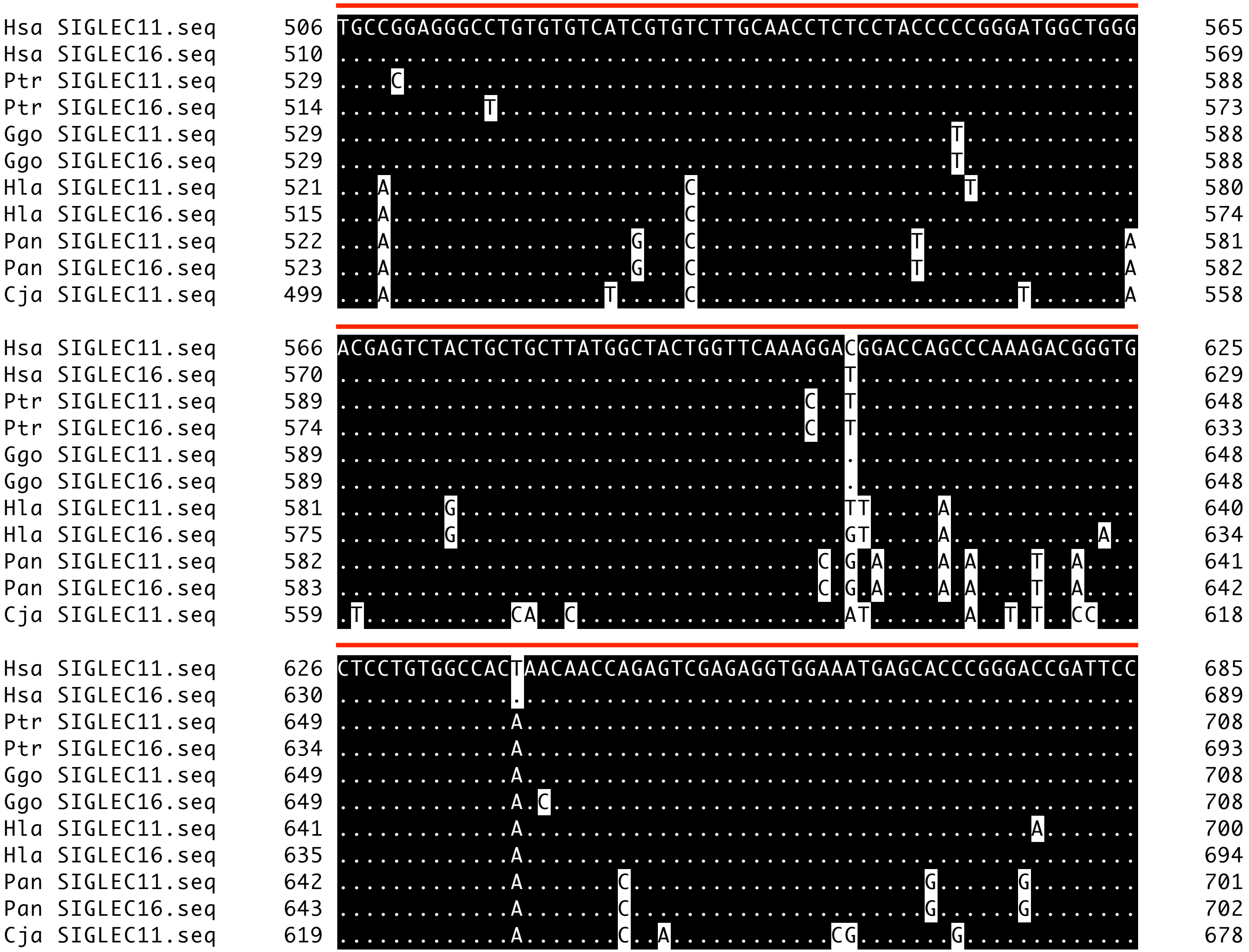
Hsa SIGLEC11.seq 506 TGCCGGAGGGCCTGTGTGTCATCGTGTCTTGCAACCTCTCCTACCCCCGGGATGGCTGGG 565
Hsa SIGLEC16.seq 510 569
Ptr SIGLEC11.seq 529 ....C... 588
Ptr SIGLEC16.seq 514 ....T.. 573
Ggo SIGLEC11.seq 529 ....T............. 588
Ggo SIGLEC16.seq 529 ....T............. 588
Hla SIGLEC11.seq 521 ...A......................C....................T............ 580
Hla SIGLEC16.seq 515 ...A......................C................................. 574
Pan SIGLEC11.seq 522 ...A..................G...C................T...............A 581
Pan SIGLEC16.seq 523 ...A..................G...C................T...............A 582
Cja SIGLEC11.seq 499 ...A................T.....C........................T.......A 558
Hsa SIGLEC11.seq 566 ACGAGTCTACTGCTGCTTATGGCTACTGGTTCAAAGGACGGACCAGCCCAAAGACGGGTG 625
Hsa SIGLEC16.seq 570 ....T..................... 629
Ptr SIGLEC11.seq 589 ...................................C..T..................... 648
Ptr SIGLEC16.seq 574 ...................................C..T..................... 633
Ggo SIGLEC11.seq 589 648
Ggo SIGLEC16.seq 589 648
Hla SIGLEC11.seq 581 ........G.............................TT.....A.............. 640
Hla SIGLEC16.seq 575 ........G.............................GT.....A...........A.. 634
Pan SIGLEC11.seq 582 ....................................C.G.A....A.A....T..A.... 641
Pan SIGLEC16.seq 583 ....................................C.G.A....A.A....T..A.... 642
Cja SIGLEC11.seq 559 .T...........CA..C....................AT.......A..T.T..CC... 618
Hsa SIGLEC11.seq 626 CTCCTGTGGCCACTAACAACCAGAGTCGAGAGGTGGAAATGAGCACCCGGGACCGATTCC 685
Hsa SIGLEC16.seq 630 689
Ptr SIGLEC11.seq 649 ....A.. 708
Ptr SIGLEC16.seq 634 ....A.. 693
Ggo SIGLEC11.seq 649 ....A.. 708
Ggo SIGLEC16.seq 649 .............A.C.. 708
Hla SIGLEC11.seq 641 .............A......................................A....... 700
Hla SIGLEC16.seq 635 ....A.. 694
Pan SIGLEC11.seq 642 .............A.......C......................G......G........ 701
Pan SIGLEC16.seq 643 .............A.......C......................G......G........ 702
Cja SIGLEC11.seq 619 .............A.......C..A............CG.......G............. 678

```
Hsa SIGLEC11.seq  686 AGCTCACTGGGGATCCCGGCAAAGGGAGCTGCTCCTTGGTGATCAGAGACGCGCAGAGGG 745
Hsa SIGLEC16.seq  690 ............................................................ 749
Ptr SIGLEC11.seq  709 ............................................................ 768
Ptr SIGLEC16.seq  694 ......................G..................................... 753
Ggo SIGLEC11.seq  709 ......................G.............................T....... 768
Ggo SIGLEC16.seq  709 ......................G.............................T....... 768
Hla SIGLEC11.seq  701 ......................G..................................... 760
Hla SIGLEC16.seq  695 ......................G..................................... 754
Pan SIGLEC11.seq  702 ......................G..................................... 761
Pan SIGLEC16.seq  703 .................A....G..................................... 762
Cja SIGLEC11.seq  679 ......TG...T.....A....G.............................T....T.. 738

Hsa SIGLEC11.seq  746 AGGATGAGGCATGGTACTTCTTTCGGGTGGAGAGAGGAAGCCGTGTGAGACATAGTTTCC 805
Hsa SIGLEC16.seq  750 ...........................................................G 809
Ptr SIGLEC11.seq  769 ............................................................ 828
Ptr SIGLEC16.seq  754 .......................T.................TA.......T...A....A 813
Ggo SIGLEC11.seq  769 .......................A...................................G 828
Ggo SIGLEC16.seq  769 .......................A...................................G 828
Hla SIGLEC11.seq  761 .........................................TA.......T...A....A 820
Hla SIGLEC16.seq  755 .........................A...............TA.......T........A 814
Pan SIGLEC11.seq  762 ...........CA...........................T.........T...A...TG 821
Pan SIGLEC16.seq  763 ...........C............................T....C....T...A...TG 822
Cja SIGLEC11.seq  739 ....CAC..G.C.............................TA.......TTC.AC...T 798

Hsa SIGLEC11.seq  806 TGAGCAATGCGTTCTTTCTAAAAGTAACAGGTATGGAATGGGGTGGGAACCCCTGCCTGT 865
Hsa SIGLEC16.seq  810 ...A....TT....----.......................................... 865
Ptr SIGLEC11.seq  829 C..A........................................................ 888
Ptr SIGLEC16.seq  814 ...A.....G.................................................. 873
Ggo SIGLEC11.seq  829 ...A........................................................ 888
Ggo SIGLEC16.seq  829 ...A........................................................ 888
Hla SIGLEC11.seq  821 ...A.....G.........C........................................ 880
Hla SIGLEC16.seq  815 ...C....AG.........C........................................ 874
Pan SIGLEC11.seq  822 ...A.G..TG....AG....G.....................A.......TG....T.C. 881
Pan SIGLEC16.seq  823 ...A.G..TG....A.....G.....................A........G......C. 882
Cja SIGLEC11.seq  799 ...A..---G....CA.....C.............................T.......C. 855
```

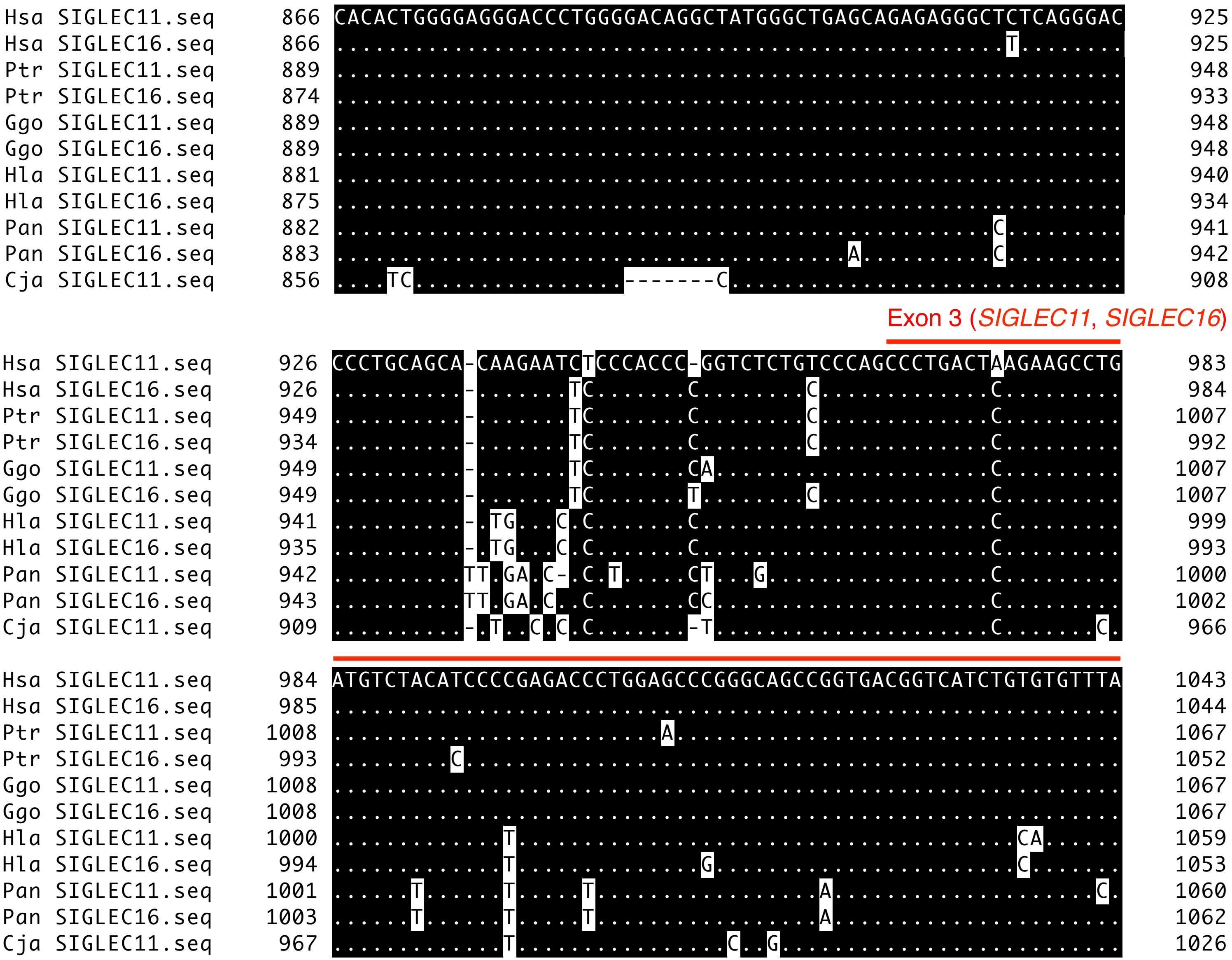

Hsa SIGLEC11.seq 866 CACACTGGGGAGGGACCCTGGGGACAGGCTATGGGCTGAGCAGAGAGGGCTCTCAGGGAC 925
Hsa SIGLEC16.seq 866 ....T........ 925
Ptr SIGLEC11.seq 889 948
Ptr SIGLEC16.seq 874 933
Ggo SIGLEC11.seq 889 948
Ggo SIGLEC16.seq 889 948
Hla SIGLEC11.seq 881 940
Hla SIGLEC16.seq 875 934
Pan SIGLEC11.seq 882 ....C......... 941
Pan SIGLEC16.seq 883 ....A..........C......... 942
Cja SIGLEC11.seq 856 ....TC................-------C.............................. 908
Exon 3 (SIGLEC11, SIGLEC16)
Hsa SIGLEC11.seq 926 CCCTGCAGCA-CAAGAATCTCCCACCC-GGTCTCTGTCCCAGCCCTGACTAAGAAGCCTG 983
Hsa SIGLEC16.seq 926 ..........-.......TC.......C........C.............C......... 984
Ptr SIGLEC11.seq 949 ..........-.......TC.......C........C.............C......... 1007
Ptr SIGLEC16.seq 934 ..........-.......TC.......C........C.............C......... 992
Ggo SIGLEC11.seq 949 ..........-.......TC.......CA.....................C......... 1007
Ggo SIGLEC16.seq 949 ..........-.......TC.......T........C.............C......... 1007
Hla SIGLEC11.seq 941 ..........-.TG...C.C.......C......................C......... 999
Hla SIGLEC16.seq 935 ..........-.TG...C.C.......C......................C......... 993
Pan SIGLEC11.seq 942 ..........TT.GA.C-.C.T.....CT...G.................C......... 1000
Pan SIGLEC16.seq 943 ..........TT.GA.C..C.......CC.....................C......... 1002
Cja SIGLEC11.seq 909 ..........-.T..C.C.C.......-T.....................C.......C. 966
Hsa SIGLEC11.seq 984 ATGTCTACATCCCCGAGACCCTGGAGCCCGGGCAGCCGGTGACGGTCATCTGTGTGTTTA 1043
Hsa SIGLEC16.seq 985 1044
Ptr SIGLEC11.seq 1008 ....A.................................. 1067
Ptr SIGLEC16.seq 993 ....C.. 1052
Ggo SIGLEC11.seq 1008 1067
Ggo SIGLEC16.seq 1008 1067
Hla SIGLEC11.seq 1000 .............T......................................CA...... 1059
Hla SIGLEC16.seq 994 .............T..............G.......................C....... 1053
Pan SIGLEC11.seq 1001 ......T......T.....T.................A....................C. 1060
Pan SIGLEC16.seq 1003 ......T......T.....T.................A...................... 1062
Cja SIGLEC11.seq 967 .............T................C..G.......................... 1026

```
Hsa SIGLEC11.seq   1044 ACTGGGCTTTCAAGAAATGTCCAGCCCCTTCTTTCTCCTGGACGGGGGCTGCCCTCTCCC 1103
Hsa SIGLEC16.seq   1045 ............................................................ 1104
Ptr SIGLEC11.seq   1068 ............................................................ 1127
Ptr SIGLEC16.seq   1053 ............................................................ 1112
Ggo SIGLEC11.seq   1068 ............................................................ 1127
Ggo SIGLEC16.seq   1068 ............................................................ 1127
Hla SIGLEC11.seq   1060 ............................................................ 1119
Hla SIGLEC16.seq   1054 ............................................................ 1113
Pan SIGLEC11.seq   1061 .......C.CTG..G..........G.....................A...........T 1120
Pan SIGLEC16.seq   1063 .......C..TG..G..........G.....................A.........T.. 1122
Cja SIGLEC11.seq   1027 .......C...G....C......CT..................................T 1086

Hsa SIGLEC11.seq   1104 CTAGAAGAACCAGACCAAGCACCTCCCACTTCTCAGTGCTCAGCTTCACGCCCAGCCCCC 1163
Hsa SIGLEC16.seq   1105 ............................................................ 1164
Ptr SIGLEC11.seq   1128 .C.........................................G.....A.......... 1187
Ptr SIGLEC16.seq   1113 .C...............................................A.......... 1172
Ggo SIGLEC11.seq   1128 .C...............................................A.....T.... 1187
Ggo SIGLEC16.seq   1128 .C...............................................A.......... 1187
Hla SIGLEC11.seq   1120 .G.A........................................................ 1179
Hla SIGLEC16.seq   1114 .G.A........................................................ 1173
Pan SIGLEC11.seq   1121 .CCA.G......A.....CG........................................ 1180
Pan SIGLEC16.seq   1123 .C.....................A.................................... 1182
Cja SIGLEC11.seq   1087 .C.................A.G.C....T..........................A.... 1146

Hsa SIGLEC11.seq   1164 AGGACCACGACACCGACCTCACCTGCCATGTGGACTTCTCCAGAAAGGGTGTGAGCGCAC 1223
Hsa SIGLEC16.seq   1165 ............................................................ 1224
Ptr SIGLEC11.seq   1188 ............................................................ 1247
Ptr SIGLEC16.seq   1173 ............................................................ 1232
Ggo SIGLEC11.seq   1188 ............................................................ 1247
Ggo SIGLEC16.seq   1188 ............................................................ 1247
Hla SIGLEC11.seq   1180 ............................C..........T......T.......C..... 1239
Hla SIGLEC16.seq   1174 ............................C..........T......T.......C..... 1233
Pan SIGLEC11.seq   1181 ..............A.............C............................... 1240
Pan SIGLEC16.seq   1183 ..............A............................................. 1242
Cja SIGLEC11.seq   1147 ..............A..............................C.............. 1206
```

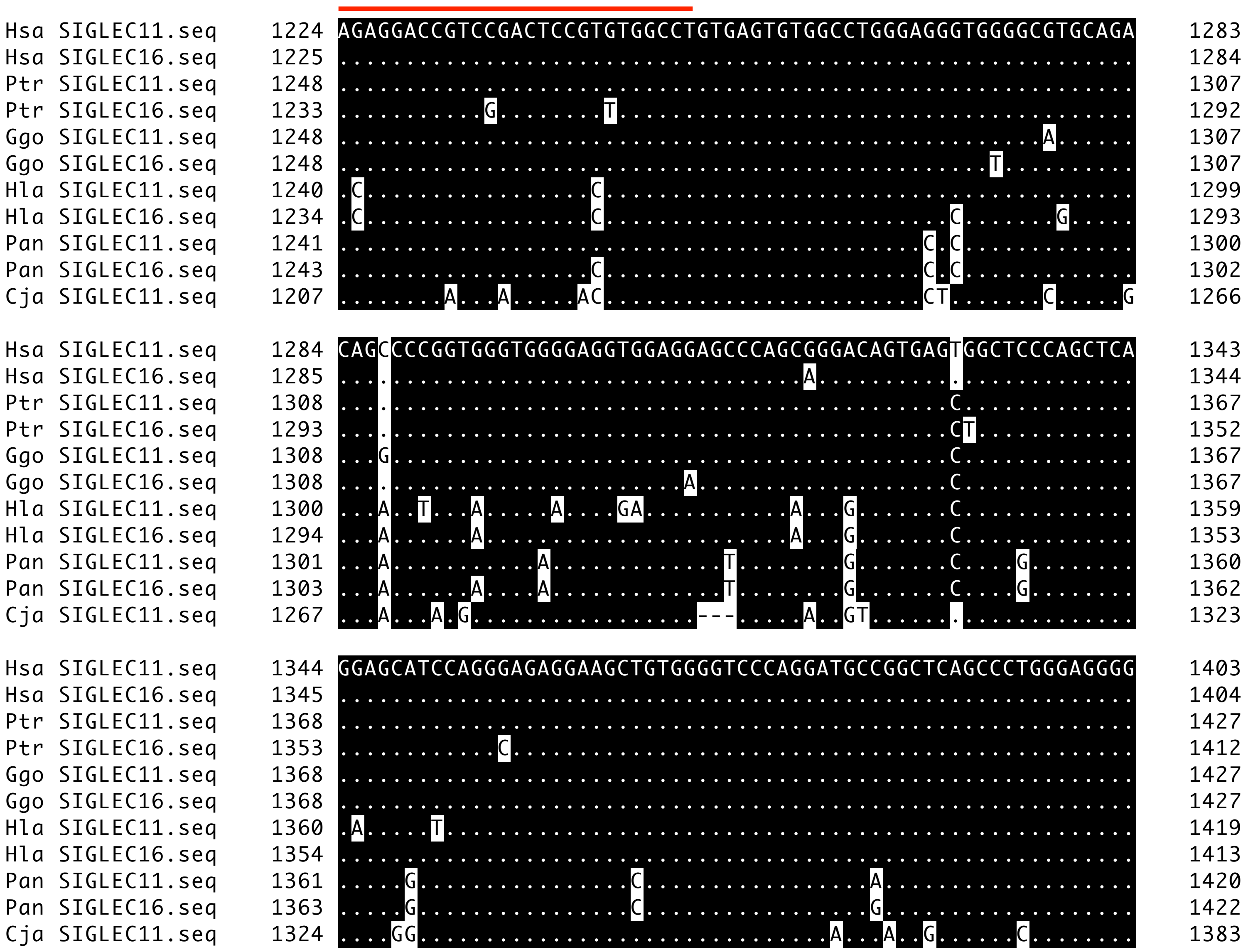
Hsa SIGLEC11.seq 1224 AGAGGACCGTCCGACTCCGTGTGGCCTGTGAGTGTGGCCTGGGAGGGTGGGGCGTGCAGA 1283
Hsa SIGLEC16.seq 1225 1284
Ptr SIGLEC11.seq 1248 1307
Ptr SIGLEC16.seq 1233 1292
Ggo SIGLEC11.seq 1248 1307
Ggo SIGLEC16.seq 1248 1307
Hla SIGLEC11.seq 1240 1299
Hla SIGLEC16.seq 1234 1293
Pan SIGLEC11.seq 1241 1300
Pan SIGLEC16.seq 1243 1302
Cja SIGLEC11.seq 1207 1266
Hsa SIGLEC11.seq 1284 CAGCCCCGGTGGGTGGGGAGGTGGAGGAGCCCAGCGGGACAGTGAGTGGCTCCCAGCTCA 1343
Hsa SIGLEC16.seq 1285 1344
Ptr SIGLEC11.seq 1308 1367
Ptr SIGLEC16.seq 1293 1352
Ggo SIGLEC11.seq 1308 1367
Ggo SIGLEC16.seq 1308 1367
Hla SIGLEC11.seq 1300 1359
Hla SIGLEC16.seq 1294 1353
Pan SIGLEC11.seq 1301 1360
Pan SIGLEC16.seq 1303 1362
Cja SIGLEC11.seq 1267 1323
Hsa SIGLEC11.seq 1344 GGAGCATCCAGGGAGAGGAAGCTGTGGGGTCCCAGGATGCCGGCTCAGCCCTGGGAGGGG 1403
Hsa SIGLEC16.seq 1345 1404
Ptr SIGLEC11.seq 1368 1427
Ptr SIGLEC16.seq 1353 1412
Ggo SIGLEC11.seq 1368 1427
Ggo SIGLEC16.seq 1368 1427
Hla SIGLEC11.seq 1360 1419
Hla SIGLEC16.seq 1354 1413
Pan SIGLEC11.seq 1361 1420
Pan SIGLEC16.seq 1363 1422
Cja SIGLEC11.seq 1324 1383

```
Hsa SIGLEC11.seq   1404 GATGGGAATGGCGTCTGATCCTCTGTCCACATGTGTGAGCCCTGGAGCTGGTTGTCACTT 1463
Hsa SIGLEC16.seq   1405 ............................................................ 1464
Ptr SIGLEC11.seq   1428 .........................................................T.. 1487
Ptr SIGLEC16.seq   1413 ............................................................ 1472
Ggo SIGLEC11.seq   1428 ............A............................................T.. 1487
Ggo SIGLEC16.seq   1428 .........................................................T.. 1487
Hla SIGLEC11.seq   1420 ..........................................C..CA............. 1479
Hla SIGLEC16.seq   1414 .............................G............C..CA............. 1473
Pan SIGLEC11.seq   1421 -................G.............CCC.....G..CA.CT............. 1479
Pan SIGLEC16.seq   1423 ............A....G..........-------------------------------- 1450
Cja SIGLEC11.seq   1384 -..........T.....G.....CA.......AC........C..CA....C........ 1442

Hsa SIGLEC11.seq   1464 GTCCATCCTGGGATGTTCCCACTTTCTTTTCCCTGAGGGAGTTTTTTCCAGGTGTGAGGA 1523
Hsa SIGLEC16.seq   1465 ............................................................ 1524
Ptr SIGLEC11.seq   1488 ..................................................AC........ 1547
Ptr SIGLEC16.seq   1473 ..................................................AC........ 1532
Ggo SIGLEC11.seq   1488 .........................G.................................. 1547
Ggo SIGLEC16.seq   1488 ............................................................ 1547
Hla SIGLEC11.seq   1480 ...T........................................................ 1539
Hla SIGLEC16.seq   1474 ...T........................................................ 1533
Pan SIGLEC11.seq   1480 ...TT...........................TG....................G..... 1539
Pan SIGLEC16.seq   1450 ------------------------------------------------------------ 1450
Cja SIGLEC11.seq   1443 ...-T..TCC...G.G........................C........T...A...... 1501

                                                                Exon 4 (SIGLEC11)
Hsa SIGLEC11.seq   1524 ACAAATTGTCCCTCCCTGAAGCCAGCTCACAATCTTGTTGCAGATGCCCCCAAAGACCTT 1583
Hsa SIGLEC16.seq   1525 ............................................................ 1584
Ptr SIGLEC11.seq   1548 ............................................C............... 1607
Ptr SIGLEC16.seq   1533 ............................................C............... 1592
Ggo SIGLEC11.seq   1548 ..........T.................................C............... 1607
Ggo SIGLEC16.seq   1548 ..........T.................................C............... 1607
Hla SIGLEC11.seq   1540 ............................................C.......G...A... 1599
Hla SIGLEC16.seq   1534 ............................................C.......G...A... 1593
Pan SIGLEC11.seq   1540 .....C..G.........T.........................C.......G....... 1599
Pan SIGLEC16.seq   1450 ------------------------------------------------------------ 1450
Cja SIGLEC11.seq   1502 ..C......-T...................C.............C.......G...GG.. 1560
```

```
Hsa SIGLEC11.seq   1584 ATTATCAGCATTTCACATGACAACACGTCAGGTACTGAGGGCCTTCGGGCTGGGGCTGGG 1643
Hsa SIGLEC16.seq   1585 ............................................................ 1644
Ptr SIGLEC11.seq   1608 .................................................T.A........ 1667
Ptr SIGLEC16.seq   1593 .............................................T.............. 1652
Ggo SIGLEC11.seq   1608 .....................G...........................T.......... 1667
Ggo SIGLEC16.seq   1608 ................G.............A..................T.......A.. 1667
Hla SIGLEC11.seq   1600 ..............G.G........................................... 1659
Hla SIGLEC16.seq   1594 ..............G.G........................................... 1653
Pan SIGLEC11.seq   1600 G.............G.G..........................G.G.............. 1659
Pan SIGLEC16.seq   1450 ------------------------------------------------------------ 1450
Cja SIGLEC11.seq   1561 G.............C.G......TGA.C.......CA......A............AA.. 1620

Hsa SIGLEC11.seq   1644 CCAGTCCTCTTTAGG-GATGAAAAGGCTTCAGGGGGG--TGAGGGGATGTGGTCCTCTTT 1700
Hsa SIGLEC16.seq   1645 ...............-.....................--..................... 1701
Ptr SIGLEC11.seq   1668 ...............-...A.................--.......G............. 1724
Ptr SIGLEC16.seq   1653 ...............-.....................--..................... 1709
Ggo SIGLEC11.seq   1668 ...............-.....................--.................T... 1724
Ggo SIGLEC16.seq   1668 ...............-................A....--..................... 1724
Hla SIGLEC11.seq   1660 ...............-..............G......GG..................... 1718
Hla SIGLEC16.seq   1654 ...............-..............G......--..................... 1710
Pan SIGLEC11.seq   1660 ...............A........ATT....T.AA..--.........A..C.....CC. 1717
Pan SIGLEC16.seq   1450 ------------------------------------------------------------ 1450
Cja SIGLEC11.seq   1621 ...........A.T.G..ATG...CAT..GCAT.TA.-GCA..........C....TCC. 1679

Hsa SIGLEC11.seq   1701 GCAGCCCCCCCTCCCACCC----ATTCTCTCTCTCCACCCCCACCCTCTCTCTTTCCCTG 1756
Hsa SIGLEC16.seq   1702 ...................----..................................... 1757
Ptr SIGLEC11.seq   1725 ...........C..---------..................................... 1775
Ptr SIGLEC16.seq   1710 ..........GC.--....----........................C............ 1763
Ggo SIGLEC11.seq   1725 ......--------.C.T.CCCC..................................... 1776
Ggo SIGLEC16.seq   1725 ......T....A...C...CCCC...............T..................... 1784
Hla SIGLEC11.seq   1719 ......T...TCT.TCT.TCTC-.C...G.........T..G...........--..... 1775
Hla SIGLEC16.seq   1711 ......T...TCT.TCT.TCTC-.C...G.........T..G...........--..... 1767
Pan SIGLEC11.seq   1718 .T....T.T.TCT.TCT.TCTC-TC..........-------------.....C..TT.C 1763
Pan SIGLEC16.seq   1450 ------------------------------------------------------------ 1450
Cja SIGLEC11.seq   1680 TGGC..TT.T..G---------------------------------.......C..TA.A 1706
```

Exon 5 (*SIGLEC11*), Exon 4 (*SIGLEC16*)

```
Hsa SIGLEC11.seq  1757 TCTTCAGCCCTGGAACTCCAGGGAAACGTCATATATCTGGAAGTTCAGAAAGGCCAGTTC 1816
Hsa SIGLEC16.seq  1758 ............................................................ 1817
Ptr SIGLEC11.seq  1776 ......................A.........G........................... 1835
Ptr SIGLEC16.seq  1764 ............................................................ 1823
Ggo SIGLEC11.seq  1777 ......................A..................................... 1836
Ggo SIGLEC16.seq  1785 ......................A.........................G........... 1844
Hla SIGLEC11.seq  1776 ..........................T................................. 1835
Hla SIGLEC16.seq  1768 ..........................T................................. 1827
Pan SIGLEC11.seq  1764 GG..........A...C.....A...TC...C.C.........CC.....G......... 1823
Pan SIGLEC16.seq  1451 -------------------------------------......CC......A......G. 1473
Cja SIGLEC11.seq  1707 .A.AT.......A.G.......A...TC...C.C.........CC............... 1766

Hsa SIGLEC11.seq  1817 CTGCGGCTCCTCTGTGCTGCTGACAGCCAGCCCCCTGCCACGCTGAGCTGGGTCCTGCAG 1876
Hsa SIGLEC16.seq  1818 ............................................................ 1877
Ptr SIGLEC11.seq  1836 ...................................................A........ 1895
Ptr SIGLEC16.seq  1824 ...................................................A........ 1883
Ggo SIGLEC11.seq  1837 ............................................................ 1896
Ggo SIGLEC16.seq  1845 ............................................................ 1904
Hla SIGLEC11.seq  1836 ....A....................................................... 1895
Hla SIGLEC16.seq  1828 ............................................................ 1887
Pan SIGLEC11.seq  1824 ...................................C........................ 1883
Pan SIGLEC16.seq  1474 ...................................C........................ 1533
Cja SIGLEC11.seq  1767 ...A...............................C.................G...... 1826

Hsa SIGLEC11.seq  1877 GACAGAGTCCTCTCCTCGTCCCACCCCTGGGGCCCCAGAACCCTGGGGCTGGAGCTGCGT 1936
Hsa SIGLEC16.seq  1878 ............................................................ 1937
Ptr SIGLEC11.seq  1896 ..........................................................CC 1955
Ptr SIGLEC16.seq  1884 ..........................................................CC 1943
Ggo SIGLEC11.seq  1897 ............................................................ 1956
Ggo SIGLEC16.seq  1905 ...................................................A........ 1964
Hla SIGLEC11.seq  1896 .......................................C..................CC 1955
Hla SIGLEC16.seq  1888 .......................................C..................C. 1947
Pan SIGLEC11.seq  1884 ................T.........................................CC 1943
Pan SIGLEC16.seq  1534 ................T.........................................CC 1593
Cja SIGLEC11.seq  1827 ..T.....G.......G.......................................T.CC 1886
```

```
Hsa SIGLEC11.seq   1937 GGGGTAAGGGCCGGGGATTCAGGGCGCTACACCTGCCGAGCGGAGAACAGGCTTGGCTCC 1996
Hsa SIGLEC16.seq   1938 ............................................................ 1997
Ptr SIGLEC11.seq   1956 ..A..G.A...T................................................ 2015
Ptr SIGLEC16.seq   1944 ..A..G.A...T...............................................T 2003
Ggo SIGLEC11.seq   1957 .......A.................................................... 2016
Ggo SIGLEC16.seq   1965 .......A................T........C.......................... 2024
Hla SIGLEC11.seq   1956 .....G.A.................................A.................. 2015
Hla SIGLEC16.seq   1948 ...A.G.A................T......T.........A.................. 2007
Pan SIGLEC11.seq   1944 .....G.A.................................................... 2003
Pan SIGLEC16.seq   1594 .....G.A...T.........................A...................... 1653
Cja SIGLEC11.seq   1887 CA...G.A...............CT................A.....T............ 1946

Hsa SIGLEC11.seq   1997 CAGCAGCAAGCCCTGGACCTCTCTGTGCAGTGTGAGTGTGCCTAGCAGGGGCCTGGAGTC 2056
Hsa SIGLEC16.seq   1998 .......G.................................................... 2057
Ptr SIGLEC11.seq   2016 .......G..................................C................. 2075
Ptr SIGLEC16.seq   2004 .......G..................................C................. 2063
Ggo SIGLEC11.seq   2017 .......G..................................C................. 2076
Ggo SIGLEC16.seq   2025 .......G..................................C...C............. 2084
Hla SIGLEC11.seq   2016 ..A....G..................................C................. 2075
Hla SIGLEC16.seq   2008 .......G..................................C.........T....... 2067
Pan SIGLEC11.seq   2004 .......G..................................C.............G... 2063
Pan SIGLEC16.seq   1654 .......G..T...............................C.............G... 1713
Cja SIGLEC11.seq   1947 .......G.......A......................--..C..........C...... 2004

Hsa SIGLEC11.seq   2057 CAT-TGGGAGGGCAGA-------GGGATACAGGGGCTGGGCTCAGGGTCCCAGAGCTGAG 2108
Hsa SIGLEC16.seq   2058 ...-............-------......................T.............. 2109
Ptr SIGLEC11.seq   2076 ...-............-------..................................... 2127
Ptr SIGLEC16.seq   2064 ...-............-------..................................... 2115
Ggo SIGLEC11.seq   2077 ...-............-------..................................... 2128
Ggo SIGLEC16.seq   2085 ...-............-------..................................... 2136
Hla SIGLEC11.seq   2076 ...-..........A.-------......................T.............. 2127
Hla SIGLEC16.seq   2068 ...-..........A.-------......................T.............. 2119
Pan SIGLEC11.seq   2064 ...-C...........ACGGGTG.....G.....A......................... 2122
Pan SIGLEC16.seq   1714 ...-C...........ACGGGTG.....G............................... 1772
Cja SIGLEC11.seq   2005 ...C..A........GACGGGTG....C................................ 2064
```

Ac/Ac'

```
Hsa SIGLEC11.seq   2109 GGGGTCTTGAACCCCAGGCCTCGGGGACTGACCTTCTT----------------------   2146
Hsa SIGLEC16.seq   2110 ......................................----------------------   2147
Ptr SIGLEC11.seq   2128 ...AC..AA.G.......T...A........TA.....----------------------   2165
Ptr SIGLEC16.seq   2116 ......................................----------------------   2153
Ggo SIGLEC11.seq   2129 ...AC..AA.G.......T...A........TA.....----------------------   2166
Ggo SIGLEC16.seq   2137 ...................................-------------------------   2171
Hla SIGLEC11.seq   2128 ....C..A..GT.................A........----------------------   2165
Hla SIGLEC16.seq   2120 ....C..A..GT.....C..........CA........----------------------   2157
Pan SIGLEC11.seq   2123 .......A..GGG.....T...A.........A.....TTTTTTGGTGTTTTTTTGAGAG   2182
Pan SIGLEC16.seq   1773 ...A...G..G...........A............-------------------------   1807
Cja SIGLEC11.seq   2065 CA.AC..G...........A......CA........----------------------   2102

Hsa SIGLEC11.seq   2146 ------------------------------------------------------------   2146
Hsa SIGLEC16.seq   2147 ------------------------------------------------------------   2147
Ptr SIGLEC11.seq   2165 ------------------------------------------------------------   2165
Ptr SIGLEC16.seq   2153 ------------------------------------------------------------   2153
Ggo SIGLEC11.seq   2166 ------------------------------------------------------------   2166
Ggo SIGLEC16.seq   2171 ------------------------------------------------------------   2171
Hla SIGLEC11.seq   2165 ------------------------------------------------------------   2165
Hla SIGLEC16.seq   2157 ------------------------------------------------------------   2157
Pan SIGLEC11.seq   2183 CAGAGTCTTGCTCTGTCTCTCAGGCTGGAGTGCAGTGGCCGGATCTCAGCTCACTGCAAG   2242
Pan SIGLEC16.seq   1807 ------------------------------------------------------------   1807
Cja SIGLEC11.seq   2102 ------------------------------------------------------------   2102

Hsa SIGLEC11.seq   2146 ------------------------------------------------------------   2146
Hsa SIGLEC16.seq   2147 ------------------------------------------------------------   2147
Ptr SIGLEC11.seq   2165 ------------------------------------------------------------   2165
Ptr SIGLEC16.seq   2153 ------------------------------------------------------------   2153
Ggo SIGLEC11.seq   2166 ------------------------------------------------------------   2166
Ggo SIGLEC16.seq   2171 ------------------------------------------------------------   2171
Hla SIGLEC11.seq   2165 ------------------------------------------------------------   2165
Hla SIGLEC16.seq   2157 ------------------------------------------------------------   2157
Pan SIGLEC11.seq   2243 CTCCGCCTCCCGGGTTTACGCCATTCTCCTGCCTCAGCCTCCCCAGTAGCTGGGACTACA   2302
Pan SIGLEC16.seq   1807 ------------------------------------------------------------   1807
Cja SIGLEC11.seq   2102 ------------------------------------------------------------   2102
```

```
Hsa SIGLEC11.seq    2146 ------------------------------------------------------------    2146
Hsa SIGLEC16.seq    2147 ------------------------------------------------------------    2147
Ptr SIGLEC11.seq    2165 ------------------------------------------------------------    2165
Ptr SIGLEC16.seq    2153 ------------------------------------------------------------    2153
Ggo SIGLEC11.seq    2166 ------------------------------------------------------------    2166
Ggo SIGLEC16.seq    2171 ------------------------------------------------------------    2171
Hla SIGLEC11.seq    2165 ------------------------------------------------------------    2165
Hla SIGLEC16.seq    2157 ------------------------------------------------------------    2157
Pan SIGLEC11.seq    2303 GGCGCCCGCCACCTCTCCTGGCTGATTTTTGTATTTTTAGTAGAGACGGGGTTTCACC    2362
Pan SIGLEC16.seq    1807 ------------------------------------------------------------    1807
Cja SIGLEC11.seq    2102 ------------------------------------------------------------    2102

Hsa SIGLEC11.seq    2146 ------------------------------------------------------------    2146
Hsa SIGLEC16.seq    2147 ------------------------------------------------------------    2147
Ptr SIGLEC11.seq    2165 ------------------------------------------------------------    2165
Ptr SIGLEC16.seq    2153 ------------------------------------------------------------    2153
Ggo SIGLEC11.seq    2166 ------------------------------------------------------------    2166
Ggo SIGLEC16.seq    2171 ------------------------------------------------------------    2171
Hla SIGLEC11.seq    2165 ------------------------------------------------------------    2165
Hla SIGLEC16.seq    2157 ------------------------------------------------------------    2157
Pan SIGLEC11.seq    2363 GTGTTAGCCAGGATGGTCTCGATCTCCTGACCTCGTGATCCGCCCGTCTCGGCCTCCCAA    2422
Pan SIGLEC16.seq    1807 ------------------------------------------------------------    1807
Cja SIGLEC11.seq    2102 ------------------------------------------------------------    2102

Hsa SIGLEC11.seq    2147 ACCTGTGTAGACCCTCATGCAGTTTGTGTCTGGGACTCAGTGGGTGATTCTGCCCTGCCC    2206
Hsa SIGLEC16.seq    2148 ............................................................    2207
Ptr SIGLEC11.seq    2166 .....................................................A......    2225
Ptr SIGLEC16.seq    2154 .....................................................A......    2213
Ggo SIGLEC11.seq    2167 .....................................................A......    2226
Ggo SIGLEC16.seq    2172 .....................................................A......    2231
Hla SIGLEC11.seq    2166 .................G..........................................    2225
Hla SIGLEC16.seq    2158 ......A..........G..........................................    2217
Pan SIGLEC11.seq    2423 .GTGC..GGATTA.AGGCTTGAGCCACCGTGCCCT..GGT..CAA..AAT...A......    2482
Pan SIGLEC16.seq    1808 ..................................G..................A......    1867
Cja SIGLEC11.seq    2103 ............--..G......G.............................TG.....    2160
```

```
Hsa SIGLEC11.seq   2207  TTCTATCCCACCCACTTCCCCCACCTCAGTGTCC-AGGATAGTTCCCTTTACCCAGAGGG   2265
Hsa SIGLEC16.seq   2208  ..............................C...-....CGC........G.........   2266
Ptr SIGLEC11.seq   2226  ..................................-.....G.........G.........   2284
Ptr SIGLEC16.seq   2214  ..................................-....CGC........G....C....   2272
Ggo SIGLEC11.seq   2227  ..............................C...-....CGC........G.........   2285
Ggo SIGLEC16.seq   2232  ..............................C...-.....GC...............A..   2290
Hla SIGLEC11.seq   2226  ....T................A............-....CGC........GA...C....   2284
Hla SIGLEC16.seq   2218  .........G...........A.......C....-....CGC........GA........   2276
Pan SIGLEC11.seq   2483  ..............................C...-....C.C................A.   2541
Pan SIGLEC16.seq   1868  ..............................C...-....CGC..............G...   1926
Cja SIGLEC11.seq   2161  A........C..A......G..........C...C....C....................   2220
```

Exon 6 (*SIGLEC11*), Exon 5 (*SIGLEC16*)

```
Hsa SIGLEC11.seq   2266  AAGCCCCTGGTCTGTCTAGAGCCGGTCCCCTGTCTCCATTTCAGATCCTCCAGAGAACCT   2325
Hsa SIGLEC16.seq   2267  ...T........C...............................................   2326
Ptr SIGLEC11.seq   2285  ...T........CA..............................................   2344
Ptr SIGLEC16.seq   2273  ...T........C...............................................   2332
Ggo SIGLEC11.seq   2286  ............................................................   2345
Ggo SIGLEC16.seq   2291  ...T..................T.....................................   2350
Hla SIGLEC11.seq   2285  ............CA................C.............................   2344
Hla SIGLEC16.seq   2277  ...G........CA..............................................   2336
Pan SIGLEC11.seq   2542  ..........C.CA.................C............................   2601
Pan SIGLEC16.seq   1927  ..........C.C..................C............................   1986
Cja SIGLEC11.seq   2221  ..........C.CA.........A.....--A............................   2278
```

```
Hsa SIGLEC11.seq   2326  GAGAGTGATGGTTTCCCAAGCAAACAGGACAGGTAGGAAAGGAGACAGAGGAGCCAGGGC   2385
Hsa SIGLEC16.seq   2327  ............................................................   2386
Ptr SIGLEC11.seq   2345  ............................................................   2404
Ptr SIGLEC16.seq   2333  ............................................................   2392
Ggo SIGLEC11.seq   2346  ...........C................................................   2405
Ggo SIGLEC16.seq   2351  ..........................................G.................   2410
Hla SIGLEC11.seq   2345  ..........................................G.................   2404
Hla SIGLEC16.seq   2337  ..........................................G.................   2396
Pan SIGLEC11.seq   2602  ..........................................G.................   2661
Pan SIGLEC16.seq   1987  ...T......................A...............G.................   2046
Cja SIGLEC11.seq   2279  ....................................C.....G..............C..   2338
```

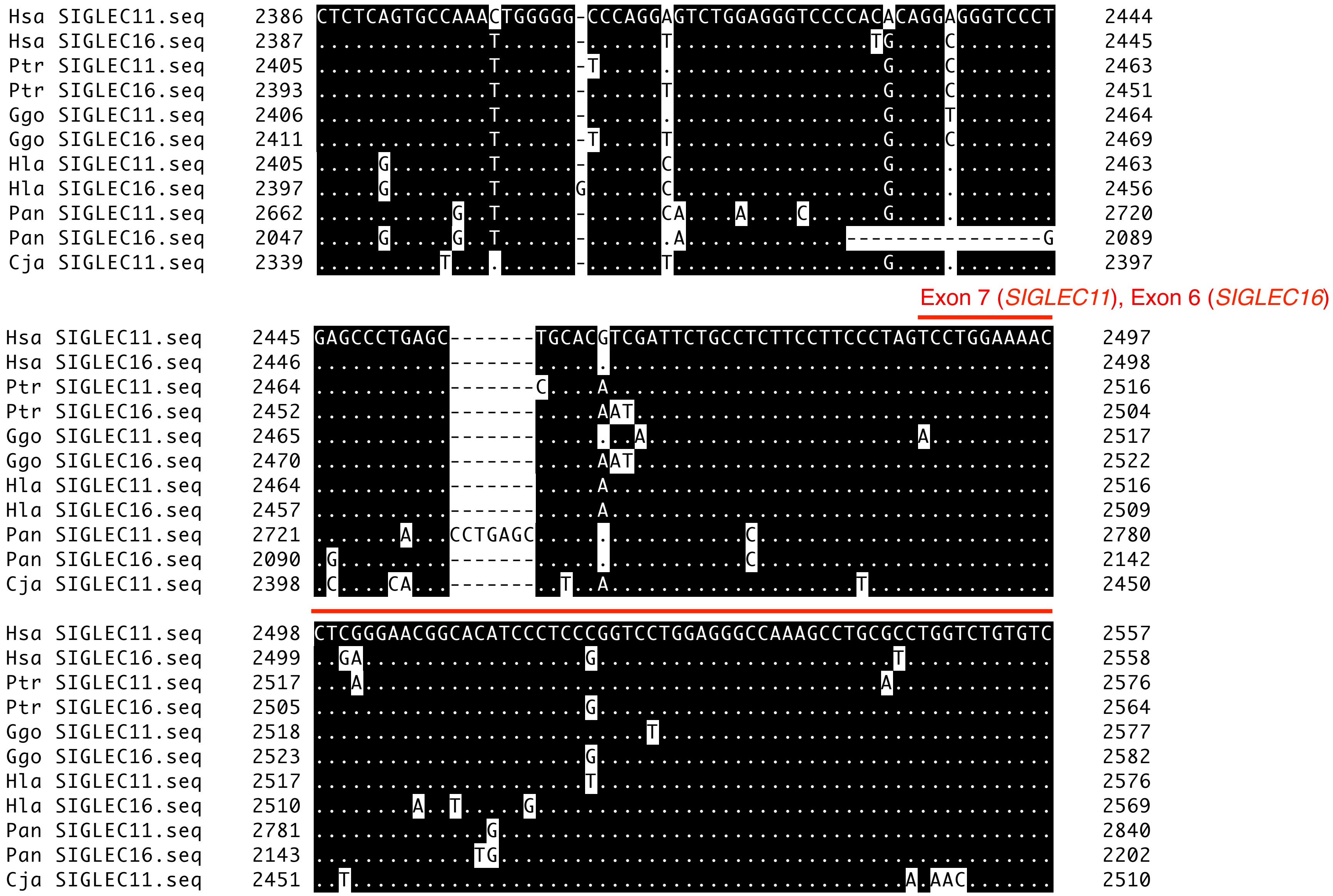
Hsa SIGLEC11.seq 2386 CTCTCAGTGCCAAACTGGGGG-CCCAGGAGTCTGGAGGGTCCCCACACAGGAGGGTCCCT 2444
Hsa SIGLEC16.seq
Ptr SIGLEC11.seq
Ptr SIGLEC16.seq
Ggo SIGLEC11.seq
Ggo SIGLEC16.seq
Hla SIGLEC11.seq
Hla SIGLEC16.seq
Pan SIGLEC11.seq
Pan SIGLEC16.seq
Cja SIGLEC11.seq
Exon 7 (SIGLEC11), Exon 6 (SIGLEC16)
Hsa SIGLEC11.seq 2445 GAGCCCTGAGC-------TGCACGTCGATTCTGCCTCTTCCTTCCCTAGTCCTGGAAAAC 2497
Hsa SIGLEC11.seq 2498 CTCGGGAACGGCACATCCCTCCCGGTCCTGGAGGGCCAAAGCCTGCGCCTGGTCTGTGTC 2557

```
Hsa SIGLEC11.seq   2558 ACCCACAGCAGCCCCCCAGCCAGGCTGAGCTGGACCCGGTGGGGACAGACCGTGG-GCCC   2616
Hsa SIGLEC16.seq   2559 ..A..................................................-....   2617
Ptr SIGLEC11.seq   2577 .......................................................-....   2635
Ptr SIGLEC16.seq   2565 ....................................T..G...A...........-....   2623
Ggo SIGLEC11.seq   2578 .......................................................-....   2636
Ggo SIGLEC16.seq   2583 .....................................A.A.........TTC..A-....   2641
Hla SIGLEC11.seq   2577 .......................................G...............-....   2635
Hla SIGLEC16.seq   2570 ...........T....T......................G...............TT...   2629
Pan SIGLEC11.seq   2841 .....................................A.G...............-....   2899
Pan SIGLEC16.seq   2203 ...........TA........................A.G.........TTC..A-....   2261
Cja SIGLEC11.seq   2511 ...........T...........................G...........C..A-....   2569

Hsa SIGLEC11.seq   2617 CTCCCAGCCCTCAGACCCCGGGGTCCTGGAGCTGCCACCCATTCAAATGGAGCACGAAGG   2676
Hsa SIGLEC16.seq   2618 ..................T.................T.GGG...................   2677
Ptr SIGLEC11.seq   2636 ......................................A.....................   2695
Ptr SIGLEC16.seq   2624 ..................T.................T.GGG...................   2683
Ggo SIGLEC11.seq   2637 ......................................G.....................   2696
Ggo SIGLEC16.seq   2642 ....................................C.GGG...................   2701
Hla SIGLEC11.seq   2636 ..................T...................A.G...................   2695
Hla SIGLEC16.seq   2630 ..................T...........A.....T.GGG..G..G.......T.....   2689
Pan SIGLEC11.seq   2900 ..............................A.....T.GGG.............T.....   2959
Pan SIGLEC16.seq   2262 ..............................AT....T.GGG.....G.......A.....   2321
Cja SIGLEC11.seq   2570 ..T..CA.............C...............T.GGG...................   2629

                                                  Bc/Bc'
Hsa SIGLEC11.seq   2677 AGAGTTCACCTGCCACGCTCAGCACCCTCTGGGCTCCCAGCACGTCTCTCTCAGCCTCTC   2736
Hsa SIGLEC16.seq   2678 ....................G......G.............G.............T....   2737
Ptr SIGLEC11.seq   2696 ............................................................   2755
Ptr SIGLEC16.seq   2684 .............T.....TG......G.............G.............T....   2743
Ggo SIGLEC11.seq   2697 ...........................A................................   2756
Ggo SIGLEC16.seq   2702 ...................GG......G.............G.............T....   2761
Hla SIGLEC11.seq   2696 ..................CTG......G................................   2755
Hla SIGLEC16.seq   2690 ..................CTG......A.................G..............   2749
Pan SIGLEC11.seq   2960 ...........................G...............A................   3019
Pan SIGLEC16.seq   2322 ...............T...........G................................   2381
Cja SIGLEC11.seq   2630 ....C..........T...........A.....G..........................   2689
```

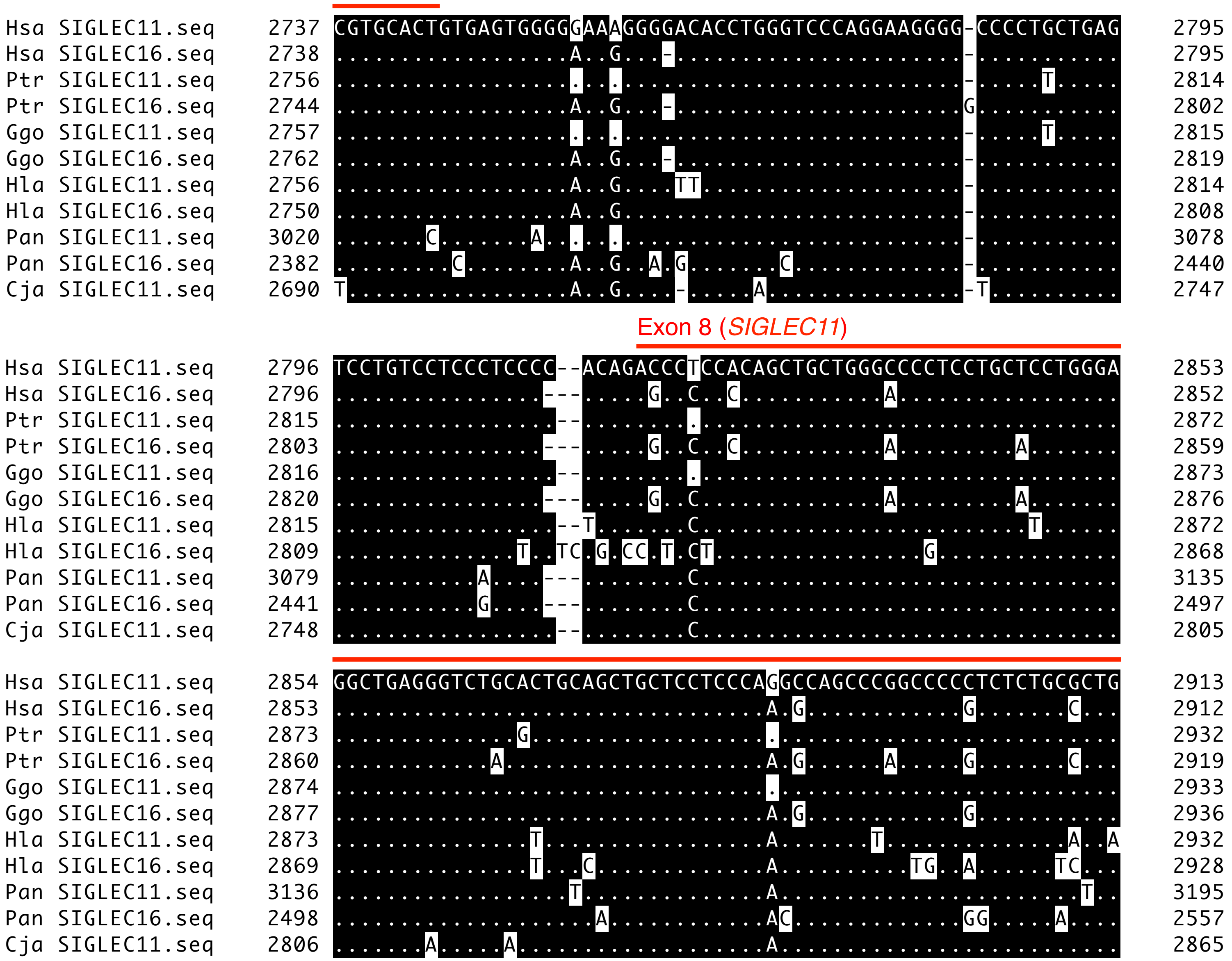
Exon 8 (*SIGLEC11*)
Hsa SIGLEC11.seq 2737 CGTGCACTGTGAGTGGGGGAAAGGGGACACCTGGGTCCCAGGAAGGGG-CCCCTGCTGAG 2795
Hsa SIGLEC11.seq 2796 TCCTGTCCTCCCTCCCC--ACAGACCCTCCACAGCTGCTGGGCCCCTCCTGCTCCTGGGA 2853
Hsa SIGLEC11.seq 2854 GGCTGAGGGTCTGCACTGCAGCTGCTCCTCCCAGGCCAGCCCGGCCCCCTCTCTGCGCTG 2913

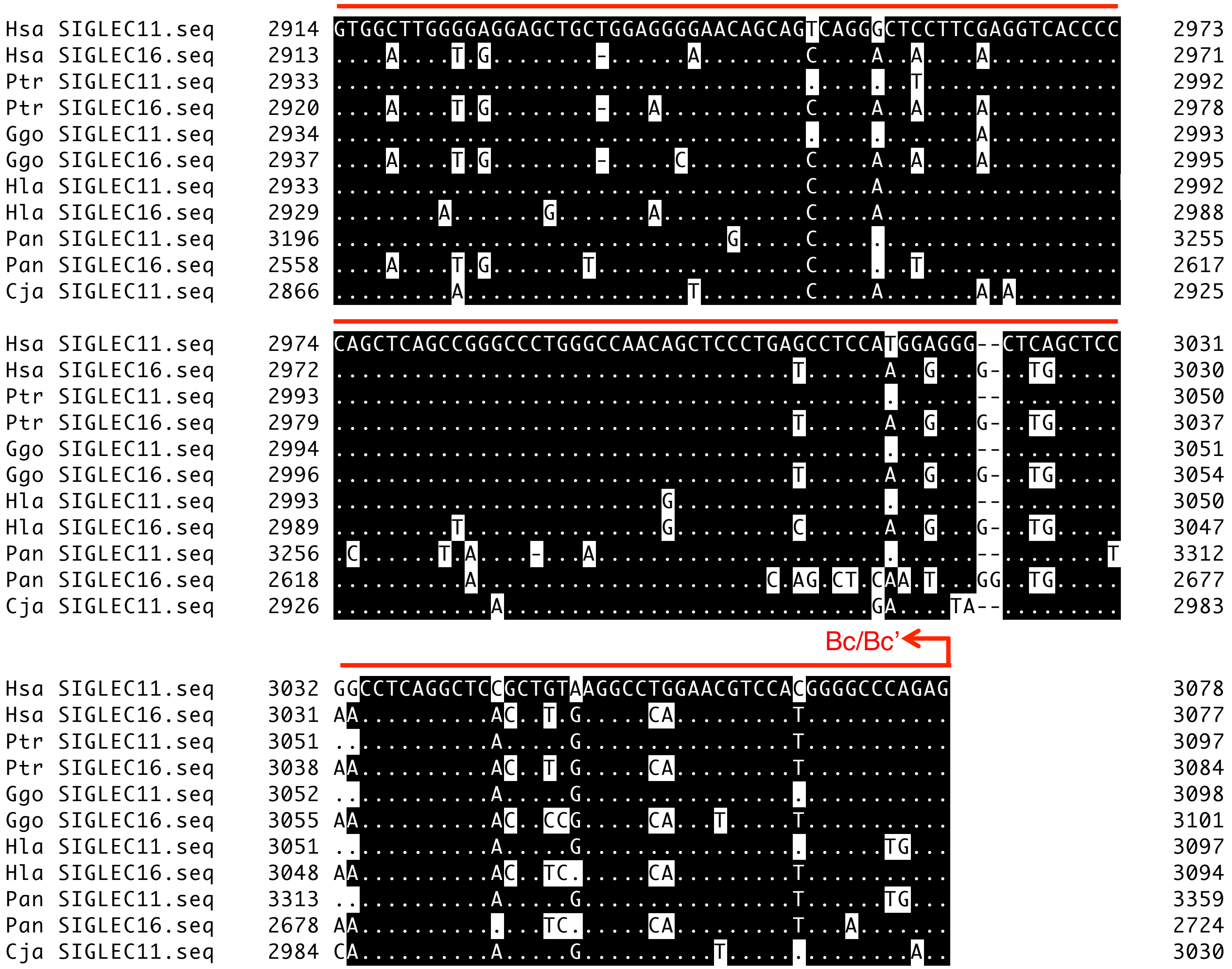
Hsa SIGLEC11.seq 2914 GTGGCTTGGGGAGGAGCTGCTGGAGGGGAACAGCAGTCAGGGCTCCTTCGAGGTCACCCC 2973
Hsa SIGLEC16.seq 2913 ....A....T.G........-......A........C....A..A....A.......... 2971
Ptr SIGLEC11.seq 2933 ....T............... 2992
Ptr SIGLEC16.seq 2920 ....A....T.G........-...A...........C....A..A....A.......... 2978
Ggo SIGLEC11.seq 2934 ....A.......... 2993
Ggo SIGLEC16.seq 2937 ....A....T.G........-.....C.........C....A..A....A.......... 2995
Hla SIGLEC11.seq 2933 ....................................C....A.................. 2992
Hla SIGLEC16.seq 2929 ........A.......G.......A...........C....A.................. 2988
Pan SIGLEC11.seq 3196 ..............................G.....C....................... 3255
Pan SIGLEC16.seq 2558 ....A....T.G.......T................C.......T............... 2617
Cja SIGLEC11.seq 2866 .........A.................T........C....A.......A.A........ 2925
Hsa SIGLEC11.seq 2974 CAGCTCAGCCGGGCCCTGGGCCAACAGCTCCCTGAGCCTCCATGGAGGG--CTCAGCTCC 3031
Hsa SIGLEC16.seq 2972 ...................................T......A..G...G-..TG..... 3030
Ptr SIGLEC11.seq 2993 ....--......... 3050
Ptr SIGLEC16.seq 2979 ...................................T......A..G...G-..TG..... 3037
Ggo SIGLEC11.seq 2994 ....--......... 3051
Ggo SIGLEC16.seq 2996 ...................................T......A..G...G-..TG..... 3054
Hla SIGLEC11.seq 2993 .........................G.......................--......... 3050
Hla SIGLEC16.seq 2989 .........T...............G.........C......A..G...G-..TG..... 3047
Pan SIGLEC11.seq 3256 .C......T.A....-...A.............................--........T 3312
Pan SIGLEC16.seq 2618 ..........A......................C.AG.CT.CAA.T...GG..TG..... 2677
Cja SIGLEC11.seq 2926 ............A............................GA....TA--......... 2983
Bc/Bc'
Hsa SIGLEC11.seq 3032 GGCCTCAGGCTCCGCTGTAAGGCCTGGAACGTCCACGGGGCCCAGAG 3078
Hsa SIGLEC16.seq 3031 AA..........AC..T.G.....CA.........T........... 3077
Ptr SIGLEC11.seq 3051 ............A.....G................T........... 3097
Ptr SIGLEC16.seq 3038 AA..........AC..T.G.....CA.........T........... 3084
Ggo SIGLEC11.seq 3052 ............A.....G............................ 3098
Ggo SIGLEC16.seq 3055 AA..........AC..CCG.....CA...T.....T........... 3101
Hla SIGLEC11.seq 3051 ............A.....G.......................TG... 3097
Hla SIGLEC16.seq 3048 AA..........AC..TC......CA.........T........... 3094
Pan SIGLEC11.seq 3313 ............A.....G................T......TG... 3359
Pan SIGLEC16.seq 2678 AA.........,...TC......CA.........T...A....... 2724
Cja SIGLEC11.seq 2984 CA..........A.....G..........T..............A.. 3030
