# Supplementary figures and images for: Coevolution of Siglec-11 and Siglec-16 via gene conversion in primates
Source: BMC Evol Biol. 2017 Nov 23;17:228. doi: 10.1186/s12862-017-1075-z (PMC5701461; doi:10.1186/s12862-017-1075-z)

Figure S2 Hayakawa et al.

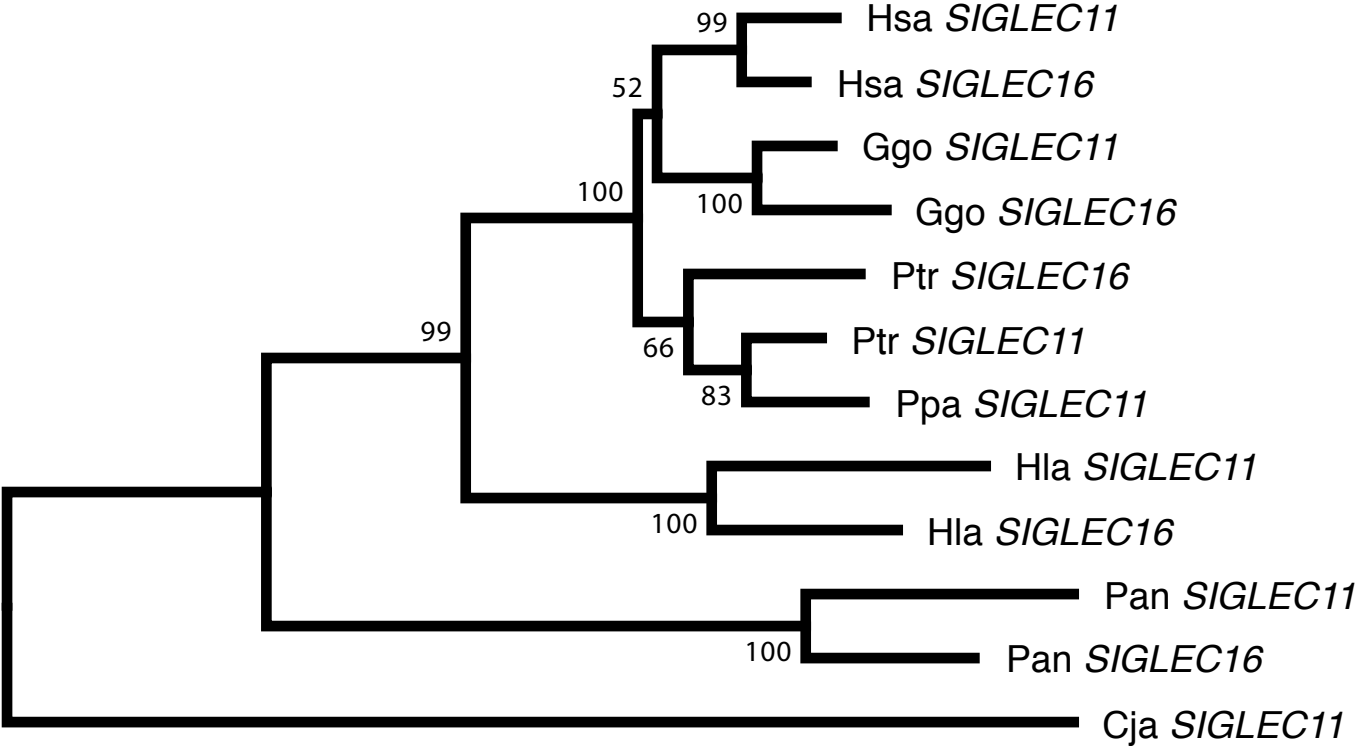

0.01

Supplement: Supplementary file 2 — Phylogenetic relationships of the A/A’ regions of SIGLEC11 and SIGLEC16. The partial sequence of bonobo SIGLEC11 was obtained previously (GenBank accession no. AB211392; [11]) and used in the tree construction. The tree topology is identical to that shown in Fig. 2A, with the exception of genes of the genus Pan. As for the lineage of the genus Pan, bonobo SIGLEC11 is most closely related to chimpanzee SIGLEC11 but the genes of genus Pan form a cluster in the tree. This suggests that gene conversion between SIGLEC11 and SIGLEC16 occurred before the divergence of chimpanzee and bonobo in the lineage of the genus Pan. Numbers on the phylogenetic tree represent bootstrap values based on 1000 replications. Hsa, Homo sapiens; Ptr, Pan troglodytes; Ppa, Pan paniscus; Ggo, Gorilla gorilla; Hla, Hylobates lar; Pan, Papio anubis; Cja, Callithrix jacchus. (PDF 45 kb) [file 12862_2017_1075_MOESM2_ESM.pdf]

Figure S4 Hayakawa et al.

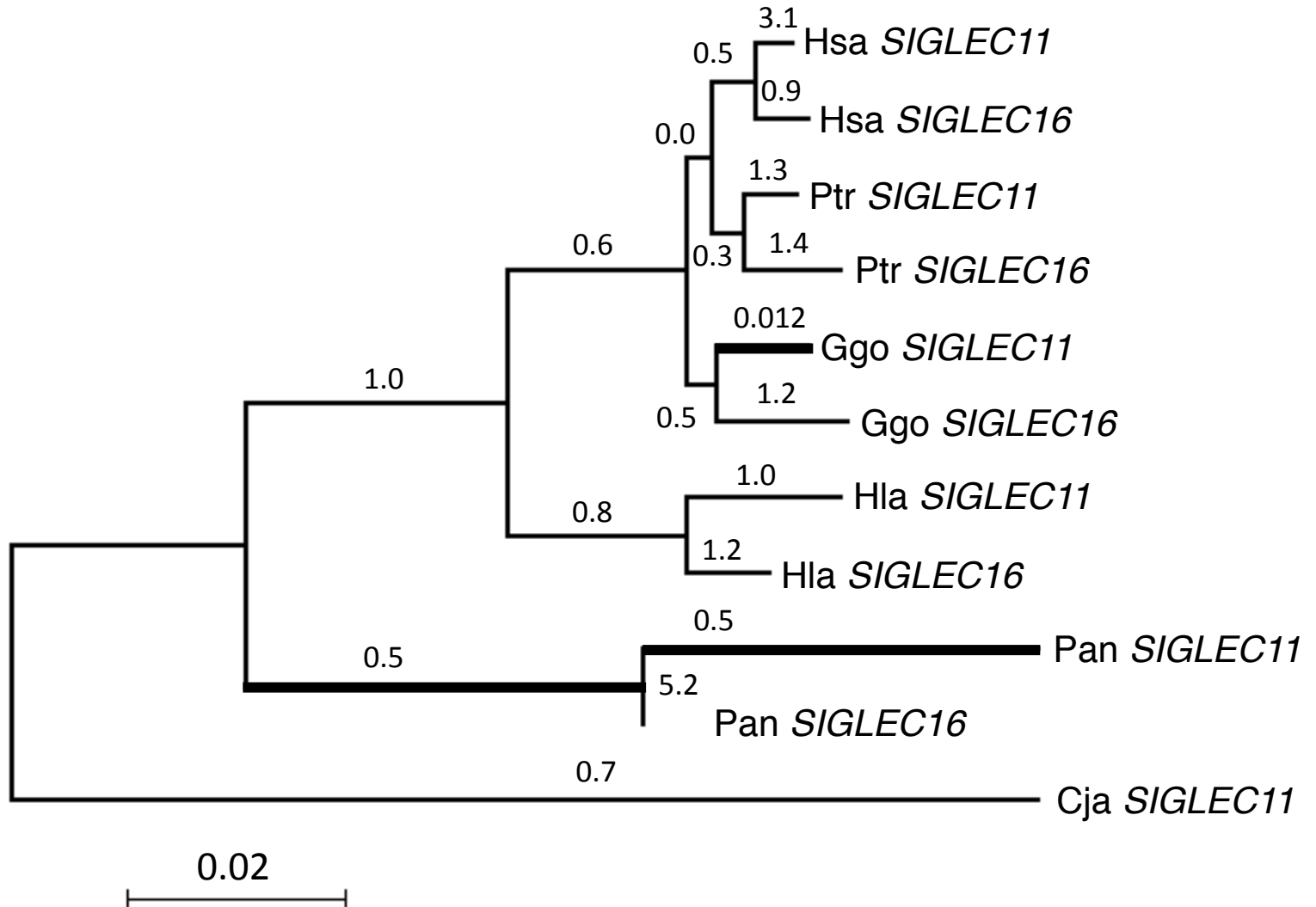

Supplement: Supplementary file 4 — Relaxed evolution of exons that underwent gene conversions. The phylogenetic tree of introns was used as a reference to examine functional constraints. The topology of the intron tree is similar to that of the Ac/Ac’ region, representing that gene conversion occurred in each primate lineage (Figure 2A). Lineage-specific ratios of nonsynonymous substitutions per site to silent substitutions per site (at both synonymous and intron sites) are shown on each branch. A significant difference between nonsynonymous substitutions per site and neutral substitutions per site is found in only three branches, one leading to gorilla SIGLEC11, one leading to baboon SIGLEC11, and the other leading to two baboon genes (P<0.02, Z-test). These branches are represented by bold lines. Hsa, Homo sapiens; Ptr, Pan troglodytes; Ggo, Gorilla gorilla; Hla, Hylobates lar; Pan, Papio anubis; Cja, Callithrix jacchus. (PDF 40 kb) [file 12862_2017_1075_MOESM4_ESM.pdf]

Figure S5 Hayakawa et al.

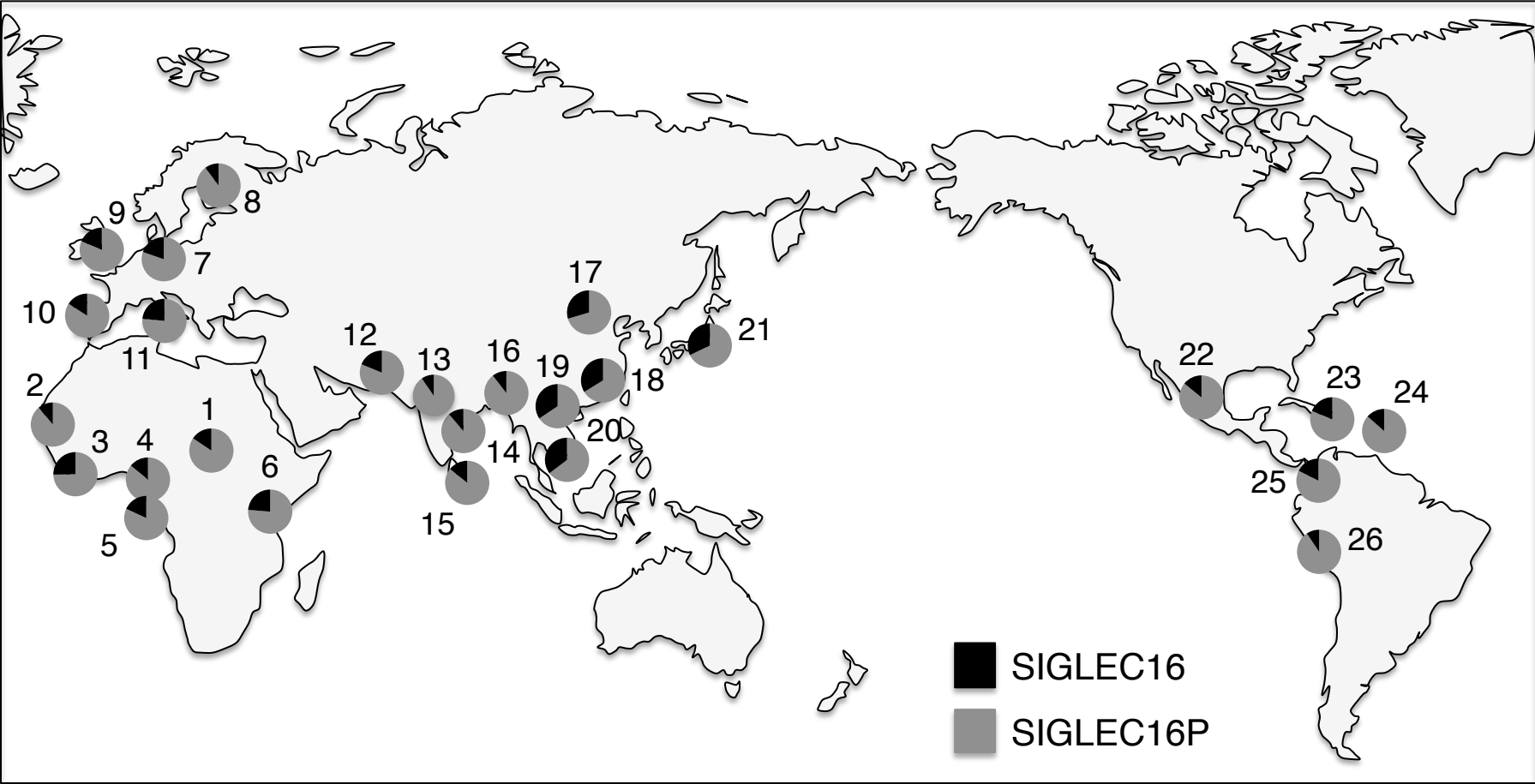

Supplement: Supplementary file 5 — Distribution of SIGLEC16 and SIGLEC16P alleles in human populations. Pie charts represent the proportion of each allele type by geographic regions. The frequencies at rs12984584 are used as a representative in this figure. 1, Americans of African ancestry (USA); 2, Gambian in Western Divisions (The Gambia); 3, Mende (Sierra Leone); 4, Esan (Nigeria); 5, Yoruba in Ibadan (Nigeria); 6, Luhya in Webuye (Kenya); 7, Utah residents (CEPH) with Northern and Western European ancestry; 8, Finnish (Finland); 9, British in England and Scotland; 10, Iberian population (Spain); 11, Tuscans (Italy); 12, Punjabi from Lahore (Pakistan); 13, Gujarati Indian from Houston (USA); 14, Indian Telugu from the UK; 15, Sri Lankan Tamil from the UK; 16, Bengali (Bangladesh); 17, Han Chinese in Bejing (China); 18, Southern Han Chinese (China); 19, Chinese Dai in Xishuangbanna (China); 20, Kinh in Ho Chi Minh City (Vietnam); 21, Japanese in Tokyo (Japan); 22, Mexican ancestry from Los Angeles (USA); 23, Puerto Ricans (Puerto Rico); 24, African Caribbeans (Barbados); 25, Colombians from Medellin (Colombia); and 26, Peruvians from Lima (Peru). Original genotyping data were obtained from the website of the 1000 Genomes Project (http://browser.1000genomes.org/index.html). (PDF 463 kb) [file 12862_2017_1075_MOESM5_ESM.pdf]
