# Supplementary material for: Coevolution of Siglec-11 and Siglec-16 via gene conversion in primates
Source: BMC Evol Biol. 2017 Nov 23;17:228. doi: 10.1186/s12862-017-1075-z (PMC5701461; doi:10.1186/s12862-017-1075-z)
Supplement: Supplementary file 3 — Alignment of amino acid sequences of Siglec-11 and Siglec-16 proteins in primates. Amino acid sequences of primate Siglec-11 and Siglec-16 (signal peptide + first and second immunoglobulin-like domains) were aligned by ClustalO. The amino acid position fully conserved among all aligned Siglec-11 and Siglec-16 is marked with asterisk (*), and the position conserved within groups of strongly or weakly similar amino acids (based on the properties of side chain) is marked with colon (:) or period (.), respectively. Functional human Siglec-16 sequence was used for the alignment. Hsa: Homo sapiens; Ptr: Pan troglodytes; Ggo: Gorilla gorilla; Hla: Hylobates lar; Pan: Papio anubis; Cja: Callithrix jacchus. Residues important for sialic acid recognition are marked with colored squares. Because the atomic level structure of Siglec-11/-16 is not available at present, amino acid residues known to be important for glycan recognition by Siglecs in common (based on the atomic level structures of Siglec-1, -2, -4, -5, and -7 in complex with respective ligand) are indicated. Red square: essential arginine residue interacting with the carboxyl group of sialic acid; Orange squares: aromatic amino acid residues involved in the coordination of sialic acid. (PDF 36 kb) [file 12862_2017_1075_MOESM3_ESM.pdf]

Figure S3 Hayakawa et al.

```
Hsa_Siglec-11   -----MLLLPLLLPVLGAGSLNKDPSYS--LQVQRQVPVPEGLCVIVSCNLSYPRDGWDE
Hsa_Siglec-16   -----MLLLPLLLPVLGAGSLNKDPSYS--LQVQRQVPVPEGLCVIVSCNLSYPRDGWDE
Ptr_Siglec-11   MLLLPLLLLPLLLPMLGAGSLNKDPSYS--LQVQRQVTVPEGLCVIVSCNLSYPRDGWDE
Ptr_Siglec-16   -----MLLLPLLLPVLGAGSLNKDPSYS--LQVQRQVPVPEGLCVIVSCNLSYPRDGWDE
Ggo_Siglec-11   MLLLPLLLLPLLLPVLGAGSLNKDPSYS--LQVQRQVPVPEGLCVIVSCNLSYPRDGWDE
Ggo_Siglec-16   MLLLPLLLLPLLLPVLAVGSLNKDPSYS--LQVQRQVPVPEGLCVIVSCNLSYPRDGWDE
Hla_Siglec-11   ----MLLLLPLLLPVLGAGFLNKDPSYS--LQVQRQVTVQEGLCVIVPCNLSYPWDGWDE
Hla_Siglec-16   -----MLLLPLLLPVLGAGSLNKDPSYS--LQVQRQVTVQEGLCVIVPCNLSYPRDGWDE
Pan_Siglec-11   -----MLLLPLLLPVLGAGSLNKDPSYSPSXQVQRLVTVQEGLCVMVPCNLSYPRDGWNE
Pan_Siglec-16   -----MLLLPLLLPVLGAGSLNKDPSYSPSLQVQRLVTVQEGLCVMVPCNLSYPRDGWNE
Cja_Siglec-11   -----MLLLSLLLPILGEGFLNKGSSYS--LQVQKLVTVQEGLCVFVPCNLSYPRVGWNE
                     :*** ****:*. * ***. ***   ***: * * *****:* ******  **:*

Hsa_Siglec-11   STAAYGYWFKGRTSPKTGAPVATNNQSREVEMSTRDRFQLTGDPGKGSCSLVIRDAQRED
Hsa_Siglec-16   STAAYGYWFKGRTSPKTGAPVATNNQSREVAMSTRDRFQLTGDPGKGSCSLVIRDAQRED
Ptr_Siglec-11   STAAYGYWFKRWTSPKTGAPVATNNQSREVEMSTRDRFQLTGDPGKGSCSLVIRDAQRED
Ptr_Siglec-16   STAAYGYWFKRWTSPKTGAPVATNNQSREVEMSTRDRFQLTGDPGKGSCSLVIRDAQRED
Ggo_Siglec-11   STAAYGYWFKGRTSPKTGAPVATNNQSREVEMSTRDRFQLTGDPGKGSCSLVIRDAQRED
Ggo_Siglec-16   STAAYGYWFKGRTSPKTGAPVATTNQSREVEMSTRDRFQLTGDPGKGSCSLVIRDAQRED
Hla_Siglec-11   SAAAYGYWFKGLTNPKTGAPVATNNQSREVEMSTRERFQLTGDPGKGSCSLVIRDAQRED
Hla_Siglec-16   SAAAYGYWFKGVTNPKTDAPVATNNQSREVEMSTRDRFQLTGDPGKGSCSLVIRDAQRED
Pan_Siglec-11   STAAYGYWFKAGTNTNTGAPVATNNPSREVEMSARGRFQLTGDPGKGSCSLVIRDAQRED
Pan_Siglec-16   STAAYGYWFKAGTNTNTGAPVATNNPSREVEMSARGRFQLTGDPSKGSCSLVIRDAQRED
Cja_Siglec-11   STATHGYWFKGMTSTYTRAPVATNNPNREVDVSTRDRFQLMGYPSKGSCSLVIRDAQMED
                *:*::*****  *.  * *****.* .*** :*:* **** * *.************ **

Hsa_Siglec-11   EAWYFFRVERGSRVRHSFLSNAFFLKVTALTKKPDVYIPETLEPGQPVTVICVFNWAFKK
Hsa_Siglec-16   EAWYFFRVERGSRVRHSFLSNAFFLKVTALTQKPDVYIPETLEPGQPVTVICVFNWAFKK
Ptr_Siglec-11   EAWYFFRVERGSRVRHSFPNNAFFLKVTALTQKPDVYIPETLEPGQPVTVICVFNWAFKK
Ptr_Siglec-16   EAWYFFRVERGSYVRYNFMNNGFFLKVTALTQKPDVYTPETLEPGQPVTVICVFNWAFKK
Ggo_Siglec-11   EAWYFFRVERGSRVRHSFVNNAFFLKVTALTQKPDVYIPETLEPGQPVTVICVFNWAFKK
Ggo_Siglec-16   EAWYFFRVERGSRVRHSFVNNAFFLKVTALTQKPDVYIPETLEPGQPVTVICVFNWAFKK
Hla_Siglec-11   EAWYFFRVERGSYVRYNFMNNGFFLKVTALTQKPDVYIPETLEPGQPVTVICMFNWAFKK
Hla_Siglec-16   EAWYFFRVERGSYVRYSFMTNRFFLKVTALTQKPDVYIPETLEPGQPVTVICVFNWAFKK
Pan_Siglec-11   EAQYFFRVERGSRVRYNFVNDWFSLEVTALTQKPDVFIPETLEPGQPVTVICVFNWASEE
Pan_Siglec-16   EARYFFRVERGSRARYNFVNDWFILEVTALTQKPDVFIPETLEPGQPVTVICVFNWAFEE
Cja_Siglec-11   TGRYFFRVERGSYVRFNFLNR-FHLTVTALTQKPDVYIPETLEPARPVTVICVFNWAFEN
                 . ********* .*..* .  * * *****:****: ******.:******:**** ::

Hsa_Siglec-11   CPAPSFSWTGAALSPRRTRPSTSHFSVLSFTPSPQDHDTDLTCHVDFSRKGVSAQRTVRL
Hsa_Siglec-16   CPAPSFSWTGAALSPRRTRPSTSHFSVLSFTPSPQDHDTDLTCHVDFSRKGVSAQRTVRL
Ptr_Siglec-11   CPAPSFSWTGAALSPRRTRPSTSHFSVLRFTPSPQDHDTDLTCHVDFSRKGVSAQRTVRL
Ptr_Siglec-16   CPAPSFSWTGAALSPRRTRPSTSHFSVLSFTPSPQDHDTDLTCHVDFSRKGVSAQRTVGL
Ggo_Siglec-11   CPAPSFSWTGAALSPRRTRPSTSHFSVLSFTPSPQDHDTDLTCHVDFSRKGVSAQRTVRL
Ggo_Siglec-16   CPAPSFSWTGAALSPRRTRPSTSHFSVLSFTPSPQDHDTDLTCHVDFSRKGVSAQRTVRL
Hla_Siglec-11   CPAPSFSWTGAALSPKRTRPSTSHFSVLSFTPSPQDHDTDLTCHVDFFRNGVTAHRTVRL
Hla_Siglec-16   CPAPSFSWTGAALSPKRTRPSTSHFSVLSFTPSPQDHDTDLTCHVDFFRNGVTAHRTVRL
Pan_Siglec-11   CPAPSFSWTGTALSSQGTKPTTSHFSVLSFTPSPQDHDTNLTCHVDFSRKGVSAQRTVRL
Pan_Siglec-16   CPAPSFSWTGTALFPRRTRPSTTHFSVLSFTPSPQDHDTNLTCHVDFSRKGVSAQRTVRL
Cja_Siglec-11   CPLPSFSWTGAALSSRRTRPRSPHFSVLSFTPRPQDHDTNLTCHVDFSRTGVSAQRTIQL
                ** *******:**  : *:* : ***** *** ******:******* *.**:*:**: *

Hsa_Siglec-11   RVA
Hsa_Siglec-16   RVA
Ptr_Siglec-11   RVA
Ptr_Siglec-16   RLA
Ggo_Siglec-11   RVA
Ggo_Siglec-16   RVA
Hla_Siglec-11   RVA
Hla_Siglec-16   RVA
Pan_Siglec-11   RVA
Pan_Siglec-16   RVA
Cja_Siglec-11   HVA
                ::*
```
